# Functional single nucleotide polymorphisms in *CACNA2D3* and other autophagy-related genes are associated with leprosy among Brazilians

**DOI:** 10.1371/journal.pntd.0014241

**Published:** 2026-04-27

**Authors:** Isabela Espasandin, Cynthia Chester Cardoso, Thyago Leal-Calvo, Mayara Abud Mendes, Ana Carla Pereira Latini, Samuel Henrique Malcher de Castro, André Luiz Leturiondo, Ohanna Cavalcanti de Lima Bezerra, Roberta Olmo Pinheiro, Anna Maria Sales, Milton Ozório Moraes, Fernanda de Souza Gomes Kehdy

**Affiliations:** 1 Leprosy Laboratory, Oswaldo Cruz Institute/FIOCRUZ, Rio de Janeiro, Rio de Janeiro, Brazil; 2 Molecular Virology Laboratory, Department of Genetics, Federal University of Rio de Janeiro, Rio de Janeiro, Rio de Janeiro, Brazil; 3 University of California Berkeley, Innovative Genomics Institute, Berkeley, California, United States of America; 4 Research and Teaching Division, Lauro de Souza Lima Institute, Bauru, São Paulo, Brazil; 5 Laboratory of Molecular Biology, Alfredo da Matta Hospital Foundation, Manaus, Amazonas, Brazil; 6 Dalla Lana School of Public Health, University of Toronto, Toronto, Ontario, Canada; UFSJ: Universidade Federal de Sao Joao del-Rei, BRAZIL

## Abstract

**Background:**

Autophagy is a crucial host defense mechanism against intracellular pathogens, including *Mycobacterium leprae*. Genetic variants in autophagy-related genes have been associated with susceptibility to leprosy, but their functional relevance remains incompletely understood.

**Methodology/Principal findings:**

We investigate the association of single nucleotide polymorphisms (SNPs) in three genes involved in autophagy, *CACNA2D3*, *LRRK2* and *IRGM*. A total of 3,480 individuals from three Brazilian populations were included in a case-control design. We confirmed that the SNP rs1449325 in *CACNA2D3* was associated with leprosy *per se* protection in the overdominant model (ORoverdTC = 0.70; p = 0.00443) in Rio de Janeiro, which was then replicated in samples from Manaus and Rondonópolis. Curiously, CC genotype of rs1449325 was associated with leprosy *per se* risk in the recessive model in Rio de Janeiro (ORrecCC = 1.51; p = 0.00476), Manaus (ORrecCC = 3.06; p = 1.44E-07) and Rondonópolis (ORrecCC = 1.50; p = 0.0240). Data from public eQTLs databases and gene expression analysis from whole blood samples suggested increasing *CACNA2D3* expression levels with TT < CT < CC genotypes. In the literature, *CACNA2D3* mRNA levels are positively correlated with calcium influx levels. Taken together, the genetic and expression data support the hypothesis that either low or high levels of calcium leads to *M. leprae* susceptibility. Associations of SNPs in *LRRK2* and *IRGM* genes were also observed in the Rio de janeiro population, although not confirmed in replication cohorts. However, a protective effect of the *LRRK2* haplotype C/G/G/T/G (rs7308720/rs7133914/rs10878434/rs3761863/rs7962370), apparently driven by rs3761863 T allele, was observed in Rio de Janeiro (ORhap = 0.44; p = 0.0121). This allele was associated with lower levels of *LRRK2* mRNA expression in skin biopsy samples from leprosy patients, as well as in tibial nerve and fibroblast samples of healthy individuals from public databases.

**Conclusions/Significance:**

Our results highlight a dual role of calcium signaling and autophagy gene regulation in leprosy susceptibility. Variants in *CACNA2D3* and *LRRK2* modulate host response to *M. leprae* infection and represent potential targets for improved therapeutic and preventive approaches.

Leprosy is a chronic infectious disease caused by *Mycobacterium leprae* that still affects thousands of people worldwide, with Brazil having the second highest number of cases. Our body’s ability to fight this infection depends, in part, on a process called autophagy, which is a kind of cellular “clean-up” system that helps remove harmful microorganisms. In this study, we examined genetic variations in three genes involved in autophagy: *CACNA2D3*, *LRRK2*, and *IRGM*, aiming to understand their impacts on leprosy susceptibility. We analyzed the DNA of 3,480 individuals from different regions of Brazil to identify which genetic profiles were more common in people with or without leprosy. We found that variations in *CACNA2D3* that led to very high or very low levels of the protein encoded by this gene contributed to a higher risk of developing leprosy. This gene is involved in regulating calcium levels in cells, suggesting that calcium balance may influence the body’s response to *M. leprae*. We also identified that a variation in *LRRK2*, which is associated with lower levels of the protein encoded in the skin and nervous tissues, contributes to a protective effect against leprosy development. Our results help explain why some people are more resistant to leprosy than others and provide new clues for developing better prevention and treatment strategies based on the body’s own immune mechanisms.

## Introduction

Leprosy is a chronic complex infectious disease, mainly caused by the intracellular pathogen *Mycobacterium leprae* or by the recently described *M. lepromatosis*. *M. leprae* preferentially infects macrophages and Schwann cells [1]. Host genetics, as well as environmental and pathogen factors, contribute to the outcome of the disease. Results from small candidate gene studies to large-scale approaches, such as genome-wide associated studies (GWAS) and whole exome sequencing (WES), have found several single nucleotide polymorphisms (SNPs) associated with leprosy, mostly in genes involved in the cellular immune response [2–8]. The progress from infection to disease also requires the hosts to be vulnerable, mainly due to environmental factors such as no BCG vaccination, nutrient deficiency in vitamins, iron, amino acids (tryptophan), and overall life conditions (poverty) [9–11].

Macroautophagy, here called autophagy, is an evolutionary conserved lysosomal degradation pathway that is associated with the control of bacillary load in *M. leprae* - infected cells [12]. Subtle changes in specific genes contribute to the susceptibility phenotype that impairs autophagy. Consistent results from large-scale genetic studies showed that the strongest signal of association with leprosy observed so far was captured by polymorphisms in *PRKN*. These polymorphisms were detected in Vietnamese families and replicated in a case-control study on Brazilians [13,14]. Parkin, encoded by *PRKN*, is an E3 ubiquitin ligase involved in protein and organelles tagging for degradation, including phagocytosed bacteria. Moderate to high levels of parkin are necessary to induce autophagy, thus playing a protective role during infection [15–17].

Our previous studies have demonstrated that in lepromatous macrophages *M. leprae* blockades the autophagy pathway by a beclin-1-mediated mechanism and that the impairment of autophagy could be associated with the outcome of type 1 reaction in multibacillary patients [12,18]. In addition, the gene encoding 2’-5’ oligoadenylate synthetase-like (OASL) underwent the greatest upregulation and was also shown to be upregulated in *M. leprae*-infected human macrophage cell lineages, primary monocytes, and skin lesion specimens from patients with a disseminated form of leprosy. Upregulation of OASL is associated with impairment of autophagy pathway and mycobacterium survival inside host cells [19].

Therefore, the importance of autophagy in the pathophysiology of leprosy is already consolidated, thus highlighting the need to understand how polymorphisms in genes related to different regulatory pathways of this process can impact the outcome of this disease. Other genes related to autophagy and metabolism associated with leprosy and its clinical forms also were identified by GWAS such as *LACC1*, *LRRK2*, *NOD2*, among others [2–8].

*LRRK2* (leucine-rich repeat kinase 2) is involved in several cellular processes, such as: vesicular trafficking and endocytosis, protein synthesis, regulation of the immune response, inflammation, presynaptic and cytoskeleton homeostasis, among others [20,21]. It works by interacting with Parkin, increasing protein ubiquitination, regulating apoptosis and autophagy by different pathways [22,23]. *IRGM* is also involved in autophagy regulation by the interaction with several proteins, acting from the initiation to the assembly of the autophagic machinery, promoting the activation of this process. The interaction with NOD2 promotes IRGM oligomerization, which seems to be essential for its functions and biochemical properties [24–27]. The *NOD2* association with leprosy has already been replicated in different populations, reinforcing the hypothesis of a possible association of *IRGM* with this outcome [6,28,29].

The gene *CACNA2D3* was down-regulated in macrophage-derived monocytes after *M. leprae* infection [10]. This encodes a subunit of the voltage-gated Ca^2^^+^ (Cav) channel complex and is a target of miRNA-27a, which acts by inhibiting its expression. miRNA27a expression seems to be actively induced in cells infected by *M. tuberculosis*, negatively regulating autophagy and favoring its survival [30–32]. It is also known that Ca^2^^+^ decreased flux has an important role favoring tuberculosis pathogenesis, influencing autophagosomes maturation and autophagosome-lysosome fusion [33].

All together, these findings corroborate the importance of autophagy in the pathophysiology of leprosy, thus highlighting the need to understand how polymorphisms in genes related to different regulatory pathways of this process can impact the outcome of this disease. Here we have further investigated the role of genes related to autophagy in leprosy outcome and clinical forms by investigating the association of candidate SNPs located in *LRRK2*, *IRGM* and *CACNA2D3* genes. A potential functional role of the associated polymorphisms was also validated through gene expression analyses.

## Methods

### Ethics statement

All study participants have signed an informed consent, in accordance with the local ethics boards and the Brazilian National Board for Ethics in Research (Rio de Janeiro–IRB protocol–Fiocruz 151/01; Manaus– 555.620/13 and Rondonópolis–ILSL 172/09).

### Patients, samples and association study design

The association study was conducted using a stepwise replication design, including three independent case-control populations with a total of 3,480 participants. First, a case-control analysis was conducted in a discovery sample from Rio de Janeiro (RIO, southeast Brazil), followed by a replication in Manaus (MAN, Amazonas State, North Brazil), and then replication in Rondonópolis (ROO, State of Mato Grosso, Midwest Brazil). Leprosy clinical forms were determined using both the Ridley and Jopling classification method [34] and the World Health Organization guideline [35,36]. The demographic characteristics of the samples from the three populations used in the study are described in Table A in S1 Text.

A total of 1,338 were enrolled in RIO study. The cases group included 759 patients from Souza Araujo Out-Patient Unit (Fiocruz, Rio de Janeiro, Brazil) diagnosed with leprosy *per se*. Among these, 267 developed the paucibacillary (PB) form and 398 the multibacillary (MB) form. The control group was composed by 579 healthy individuals selected among bone marrow donors from the bank of the Cancer National Institute (INCA) (Rio de Janeiro-RJ, Brazil) [37]. The replication population of MAN consisted of 1,374 samples recruited at Fundação Hospitalar Alfredo da Matta (FUHAM, Manaus-AM, Brazil), with 407 cases and 967 controls. Patients were stratified as 130 PB and 277 MB, and healthy volunteers resident in the same endemic area as the cases were recruited for the control group in the same clinic (FUHAM), after a dermatological check-up [29]. The second replication population from ROO was composed of 768 individuals, including 411 cases and 357 controls. Patients from different public health institutions (primary care facilities), were divided as 96 PB and 310 MB. Healthy individuals were recruited in the same region during dermatological campaigns.

### Candidate SNPs selection

The same analytical workflow was applied to *CACNA2D3*, *IRGM*, and *LRRK2*, following the methodology previously described by Bezerra et al. [37]. We began by selecting SNPs located within ±5 kb of each gene, using genomic data from the 1000 Genomes Project phase III [38]. This dataset was filtered to include only the three main ancestral populations of Brazilians: 504 Africans (ENS, GWD, LWK, MSL, YRI), 503 Europeans (CEU, FIN, GBR, IBS, TSI), and 347 Native Americans. Allele frequencies were calculated, and SNPs were annotated using ANNOVAR [39]. In parallel, PCA was performed with EIGENSOFT 4.2 [40,41], considering the genotypes of individuals from these populations (see Figs A and B in S1 Text). Linkage disequilibrium and haplotype frequencies were assessed in Africans and Europeans using HAPLOVIEW 4.2 [42] (Figs C, D and E in S1 Text).

SNP selection involved multiple decision-making steps based on: (1) functional annotation (e.g., exonic, intergenic, UTRs, intronic); (2) SNPweight in PCA components 1 and 2; (3) MAF ≥ 5% in Africans and Europeans; (4) absence of strong LD (r² > 0.8) between preselected SNPs and differing haplotype patterns between Europeans and Africans; and (5) prior associations with leprosy, IBDs, tuberculosis and others with aspects of pathophysiology in common with leprosy. Finally, selected SNPs were queried in the GTEx database [43] to assess eQTL (expression quantitative trait locus) associations with gene expression.Table B in S1 Text summarizes the 11 selected TagSNPs located in candidate genes and their flanking regions.

### SNPs genotyping and association analyses

The DNA was extracted from whole blood samples by the salting out method [44] and all samples had their concentrations adjusted to 10–40 ng/μL. Samples were genotyped for all selected SNPs by real-time PCR with allelic discrimination using the TaqMan Genotyping Assays [45]. The genotyping reactions were prepared with a final volume of 5 μL (2.5 μL of TaqMan Genotyping Master Mix (Thermo Fisher Scientific), 0.125 μL of TaqMan primers and probes, 1.375 μL of H2O and 1 μL of DNA). Reactions were run on a StepOnePlus 2.1 real-time PCR instrument (Thermo Fisher Scientific), using standard cycling conditions for SNP genotyping. Genotype analyses were performed using StepOne Software v2.3.

Statistical analyses were run on R environment (R DEVELOPMENT CORE TEAM, 2013) using Genetics, SNPassoc, Haplo.stats and nnet packages. Genotype and allele frequencies were determined and possible deviations from the Hardy-Weinberg Equilibrium (HWE) were assessed using the chi-square test in cases and controls cohorts. Linkage disequilibrium (LD) patterns between SNPs of each target gene were determined in the control groups and represented using the coefficient r^2^. These analyses were performed in HAPLOVIEW software 4.2 program [42]. Haplotype frequencies were estimated by maximum likelihood.

The association of each SNP and haplotype with leprosy *per se* was determined using logistic regression models (codominant, dominant, recessive, superdominant and log-additive) adjusted for sex, age, and ancestry [37] (when these presented significant results from previous association analyses for such covariates with leprosy outcome). The effect estimates used were the odds ratio (OR), along with 95% confidence interval. The association between SNPs and leprosy clinical forms were determined from multinomial regression models. The aim of this was to estimate the effect of polymorphisms on leprosy susceptibility by stratifying cases by PB and MB forms, as described by Gilchrist *et al* [46]. Nominal p-values < 0.05 were considered statistically significant. To account for multiple testing, p-values were subsequently adjusted using the Benjamini–Hochberg false discovery rate (FDR) method for each population, adopting an FDR threshold of 0.05. For each SNP, FDR correction was applied considering the p-value derived from the genetic model presenting the lowest Akaike Information Criterion (AIC), thereby selecting the most parsimonious model. For multinomial analyses, FDR correction was applied separately for each clinical outcome (controls vs. PB and controls vs. MB).

### Gene expression analyses in macrophages

We retrieved results from a publicly available dataset from genome-wide gene expression pattern analyses in macrophages isolated from leprosy patients before and after live *M. leprae* infection [10]. We genotyped 16 of these patients for rs1449325 and used their recovered expression values to investigate the genotype-phenotype relationship between this SNP and *CACNA2D3* expression levels in macrophages related to *M. leprae* infection [45]. The means of expression values for *CACNA2D3* from each genotype group were compared using the Paired T test.

### cDNA synthesis, RT-PCR and statistical analyses

A total of 2.5mL of blood samples from 55 patients recruited at the FIOCRUZ were collected before starting multidrug therapy and who did not develop reactional episodes, in RNA stabilizer tubes - PAXgene (PreAnalytix, QIAGEN, USA). Of these, 34 patients were diagnosed with PB leprosy and 21 with MB (Table C in S1 Text). Total RNA from whole blood samples was extracted using the protocol recommended by the manufacturer of PAXgene extraction kit (Qiagen). Then, a total of 2.5 μg of RNA extracted was converted into cDNA in a reverse transcription reaction using 4 μL of Vilo Master Mix (Thermo Fisher Scientific) following standard manufacturer’s protocol and diluted to a final concentration of 5 ng/μL. The primers for each target gene were designed using the NCBI Primer-Blast [47], prioritizing annealing in flanking regions of introns or exon-exon junctions, to avoid gDNA amplification. We then used MFEPrimer v.3.0 [48–50] and IDT’s oligoAnalyzer (https://www.idtdna.com/calc/analyzer) to evaluate the primers for specificity and formation of dimers and hairspins. Primers designed for *CACNA2D3*, *IRGM* and *LRRK2* and the reference genes *RPL13* and *RPS16* are listed in Table D in S1 Text.

The relative expression levels of each gene were analyzed by RT-qPCR using Sybr Green assays (Thermo Fisher Scientific) in a reaction with a final volume of 10 μL (5 μL of Fast Sybr Master Mix (Thermo Fisher Scientific), 200 nM of each primer, 10 ng of cDNA and injection grade water for final volume). Thermal cycling was performed by StepOnePlus 2.1 Real-Time PCR (Thermo Fisher Scientific), according to the manufacturer’s recommendations, and raw data were exported from StepOne Software v2.3. Next, we used LinRegPCR v.2020.0 to determine the efficiency of RT-qPCR and the N0 value of each reaction [51,52]. The normalization factor (NF) for each sample was calculated from the geometric mean of the N0 values of two reference genes (*RPL13* and *RPS16*). Then, the N0 values of each reaction for all genes were normalized using NF as denominator [45].

Normalized gene expression values from 21 skin biopsies from patients with leprosy and who did not develop reactional episodes (Table C in S1 Text) were retrieved from a dataset publicly available, as described by Leal-Calvo *et al* [9]. Concomitantly, all donors of whole blood samples and skin biopsies were genotyped for the SNPs selected from the association analyses [45]. Finally, the patients´ genotypes for each SNP paired with the normalized N0 values of their respective genes were imported into GraphPad Prism 8.0.2 (GraphPad Software, San Diego, California USA). The means of normalized expression values for each gene from each genotype group of the associated SNPs were compared using the Brown-Forsythe Anova test followed by the Tamhane T2 multiple comparison test (3 groups) and the Welch’s T test (2 groups). P-values < 0.05 were considered statistically significant. Data retrieved from public databases were used to identify eQTLs in: healthy subjects [43]; monocytes stimulated with double-stranded RNA, LPS and MCP [53] and; monocytes stimulated with killed *M. leprae* [54].

## Results

First, a case-control study was carried out in the RIO population and the 11 tag SNPs selected were included. To replicate the results obtained, the SNPs significantly associated with leprosy *per se*, PB and/or MB, were tested in MAN population. Then, the SNPs whose association was replicated in this population were tested in ROO. The most frequent genotypes in each population were adopted as reference for all models analyzed. Genotype frequencies for all SNPs analyzed did not deviate from the Hardy-Weinberg equilibrium (HWE) in the control group of the studied populations.

The LD patterns for all pairwise combinations of SNPs in the same gene for the control of the studied populations, were determined by r^2^ (Fig F in S1 Text). The pairs with r^2^ ≥ 0.8 were considered in strong LD and those with 0.6 ≤ r^2^ ≤ 0.79 in moderate LD. The results indicated that tagSNPs selection for all genes were proper, since no evidence of strong LD was detected.

### SNP rs1449325 in *CACNA2D3* is associated with leprosy *per se*

**Fig 1:**
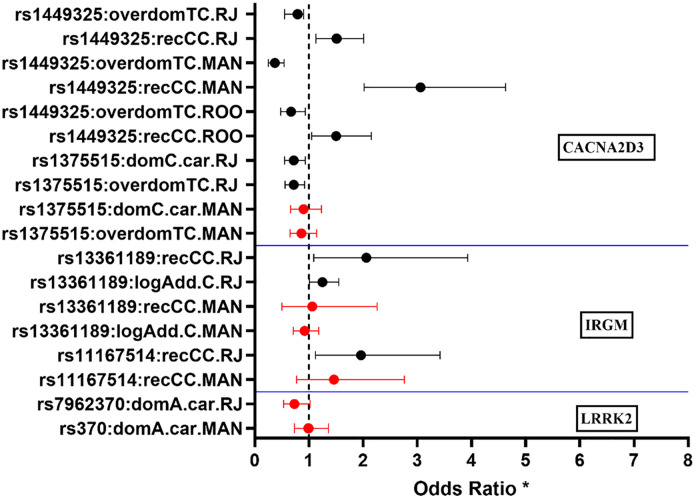
Main results of the association analysis between candidate SNPs in *CACNA2D3*, *IRGM* and *LRRK2* with the development of leprosy *per se.* Analyses were performed by logistic regression models adjusted for sex, age and ancestry. The measure of association analyzed was the odds ratio (OR) with 95% confidence intervals, with p-values less than 0.05 considered significant (black = significant; red = not significant). * The bars represent the ORs (dot) and their respective 95% confidence intervals for each SNP in different models and populations. On the SNP axis, the following representations should be considered as: domX.Car = group of genotypes carrying the minor allele x in the dominant model; recxx = homozygous genotype for the minor allele in the recessive model; overdomXx = heterozygous genotype in the overdominant model and; logAdd.x = log additive model for the minor allele.

As shown in Fig 1 and Table E in S1 Text, results of logistic regression models adjusted for sex and ancestry showed an association between SNP rs1449325 in *CACNA2D3* and leprosy *per se* under recessive (ORrecCC = 1.51; p = 0.00476), and overdominant (ORoverdTC = 0.70; p = 0.00443) models in RIO population. These risk and protective associations were replicated, in the same recessive and overdominant models, in the populations of MAN (ORrecCC = 3.06; p = 1.44E-07; ORoverdTC = 0.37; p = 1.19E-07) and ROO (ORrecCC = 1.50; p = 0.0240; ORoverdTC = 0.67; p = 0.0145). After correction for multiple testing using FDR (Table G in S1 Text) and considering the overdominant model, which presented the lowest AIC, the association of rs1449325 with leprosy *per se* remained statistically significant in RIO (FDR_p = 0.04235) and MAN (FDR_p = 5.95E-07). FDR correction was not performed for ROO, as only a single SNP was evaluated. Results of RIO cohort were similar when the covariate age was also included in the model (ORrecCC = 1.51; p = 0.03341; ORoverdTC = 0.65; p = 0.01), despite the lower statistical power (Table E in S1 Text).

The SNPs rs1375515 in *CACNA2D3*, rs13361189 and rs11167514 in *IRGM* were found to be significantly associated with leprosy *per se* in RIO, but these associations were not replicated in MAN, so these were consequently not tested in ROO (Fig 1 and Table E in S1 Text). After FDR correction (Table G in S1 Text), considering the overdominant and recessive models, which presented the lowest AIC for rs1375515, rs13361189, and rs11167514, respectively, only the association with rs1375515 in RIO remained statistically significant (FDR_p = 0.04235). The adjusted p-values for rs13361189 and rs11167514 in RIO were close to the FDR significance threshold but did not remain statistically significant.

### SNPs in *CACNA2D3* and *LRRK2* are associated with leprosy clinical forms

Association analyses of SNPs with leprosy clinical forms by multinomial logistic regression models corroborated the associations of SNPs in *CACNA2D3* and *IRGM* with leprosy *per se* detected by corresponding binomial models (Table 1 and 2). A protection association for TC genotype and a risk association for CC genotype of rs1449325 in *CACNA2D3* in the recessive and overdominant models with clinical forms PB (ORrecCC = 1.51; p = 0.0259; ORoverdTC = 0.73; p = 0.0526) and MB (ORrecCC = 1.46; p = 0.0243; ORoverdTC = 0.69; p = 0.0101) were observed in RIO. These associations were replicated in the same models with PB (ORrecCC = 2.34; p = 0.0020; ORoverdTC = 0.39; p = 0.0008) and MB (ORrecCC = 3.07; p = 7.77E-07; ORoverdTC = 0.41; p = 8.45E-05) forms in MAN and only with MB (ORrecCC = 1.49; p = 0.0386; ORoverdTC = 0.64; p = 0.0105) form in ROO. After FDR correction (Table G in S1 Text), considering the overdominant model, the associations of rs1449325 with clinical form MB remained statistically significant in RIO (FDR_p = 0.0341) and MAN (FDR_p = 4.23E-05). The association with clinical form PB remained significant after correction only in MAN (FDR_p = 0.004). We hypothesize that the lack of a statistically significant association with the PB form in RIO may be due to the smaller sample size of this cohort, which likely resulted in reduced statistical power. Notably, the direction of the effect is consistent with the associations observed for leprosy *per se* and for the MB form.

**Table 1:** Association analysis by multinomial logistic regression models for associated SNPs in *CACNA2D3*, *IRGM* and *LRRK2* with leprosy clinical forms in Rio de Janeiro (RIO).

		Rio de Janeiro						
SNP	Model	CO (%)	PB (%)	MB (%)	OR (CI 95%)^PB^	p-value^PB^	OR (CI 95%)^MB^	p-value^MB^
rs1449325	Codom.							
	TT	138 (25.5)	66 (29.1)	106 (31.5)	Ref.		Ref.	
	TC	276 (50.9)	98 (43.2)	141 (41.8)	0.84 (0.57-1.24)	0.3890	0.76 (0.54-1.07)	0.1119
	CC	128 (23.6)	63 (27.8)	90 (26.7)	1.35 (0.87-2.10)	0.1751	1.23 (0.83-1.82)	0.3112
	Dom.							
	TT	138 (25.5)	66 (29.1)	106 (31.5)	Ref.		Ref.	
	TC/ CC	404 (74.5)	161 (70.9)	231 (68.5)	1.00 (0.70-1.42)	0.9790	0.90 (0.65-1.23)	0.4952
	Rec.							
	TT/ TC	414 (76.4)	164 (72.2)	247 (73.3)	Ref.		Ref.	
	CC	128 (23.6)	63 (27.8)	90 (26.7)	1.51 (1.05-2.18)	**0.0259**	1.46 (1.05-2.03)	**0.0243**
	Overdom.							
	TT/ CC	266 (49.1)	129 (56.8)	196 (58.2)	Ref.		Ref.	
	TC	276 (50.9)	98 (43.2)	141 (41.8)	0.73 (0.53-1.00)	**0.0526**	0.69 (0.51-0.91)	**0.0101**
rs1375515	Codom.							
	TT	173 (31.1)	81 (34.6)	113 (32.1)	Ref.		Ref.	
	TC	286 (51.3)	105 (44.9)	159 (45.2)	0.67 (0.47-0.96)	**0.0289**	0.74 (0.54-1.03)	**0.0726**
	CC	98 (17.6)	48 (20.5)	80 (22.7)	0.82 (0.52-1.29)	0.3951	1.02 (0.68-1.53)	0.9129
	Dom.							
	TT	173 (31.1)	81 (34.6)	113 (32.1)	Ref.		Ref.	
	TC/ CC	384 (68.9)	153 (65.4)	239 (67.9)	0.71 (0.51-0.99)	**0.0451**	0.82 (0.60-1.10)	0.1871
	Rec.							
	TT/ TC	459 (82.4)	186 (79.5)	272 (77.3)	Ref.		Ref.	
	CC	98 (17.6)	48 (20.5)	80 (22.7)	1.04 (0.70-1.56)	0.8293	1.22 (0.86-1.73)	0.2620
	Overdom.							
	TT/ CC	271 (48.7)	129 (55.1)	193 (54.8)	Ref.		Ref.	
	TC	286 (51.3)	105 (44.9)	159 (45.2)	0.72 (0.53-0.99)	**0.0452**	0.74 (0.56-0.98)	**0.0327**
rs13361189	Codom.							
	TT	372 (66.4)	146 (58.9)	202 (54.9)	Ref.		Ref.	
	TC	174 (31.1)	87 (35.1)	142 (38.6)	1.04 (0.75-1.45)	0.8086	1.29 (0.96-1.75)	0.0911
	CC	14 (2.5)	15 (6)	24 (6.5)	2.28 (1.06-4.89)	**0.0345**	2.58 (1.26-5.26)	**0.0094**
	Dom.							
	TT	372 (66.4)	146 (58.9)	202 (54.9)	Ref.		Ref.	
	TC/ CC	188 (33.6)	102 (41.1)	166 (45.1)	1.13 (0.82-1.56)	0.4387	1.39 (1.04-1.86)	**0.0249**
	Rec.							
	TT/ TC	546 (97.5)	233 (94)	344 (93.5)	Ref.		Ref.	
	CC	14 (2.5)	15 (6)	24 (6.5)	2.26 (1.06-4.80)	**0.0341**	2.33 (1.15-4.72)	**0.0187**
	Overdom.							
	TT/ CC	386 (68.9)	161 (64.9)	226 (61.4)	Ref.		Ref.	
	TC	174 (31.1)	87 (35.1)	142 (38.6)	0.99 (0.71-1.37)	0.9419	1.21 (0.90-1.63)	0.2009
rs11167514	Codom.							
	GG	327 (60.2)	142 (57.7)	188 (50.9)	Ref.		Ref.	
	GC	197 (36.3)	84 (34.1)	152 (41.2)	0.82 (0.59-1.15)	0.2506	1.16 (0.87-1.56)	0.3212
	CC	19 (3.5)	20 (8.1)	29 (7.9)	2.14 (1.10-4.16)	**0.0251**	2.18 (1.16-4.11)	**0.0156**
	Dom.							
	GG	327 (60.2)	142 (57.7)	188 (50.9)	Ref.		Ref.	
	GC/ CC	216 (39.8)	104 (42.3)	181 (49.1)	0.94 (0.68-1.29)	0.6952	1.25 (0.94-1.66)	0.1205
	Rec.							
	GG/ GC	524 (96.5)	226 (91.9)	340 (92.1)	Ref.		Ref.	
	CC	19 (3.5)	20 (8.1)	29 (7.9)	2.32 (1.21-4.45)	**0.0114**	2.04 (1.10-3.80)	**0.0241**
	Overdom.							
	GG/ CC	346 (63.7)	162 (65.9)	217 (58.8)	Ref.		Ref.	
	GC	197 (36.3)	84 (34.1)	152 (41.2)	0.77 (0.55-1.06)	0.1131	1.08 (0.81-1.44)	0.5946
rs7962370	Codom.							
	GG	270 (71.8)	120 (72.7)	213 (77.7)	Ref.		Ref.	
	GA	98 (26.1)	40 (24.2)	54 (19.7)	0.94 (0.61-1.45)	0.7729	0.65 (0.44-0.97)	**0.0347**
	AA	8 (2.1)	5 (3)	7 (2.6)	1.04 (0.32-3.40)	0.9480	0.77 (0.26-2.27)	0.6317
	Dom.							
	GG	270 (71.8)	120 (72.7)	213 (77.7)	Ref.		Ref.	
	GA/ AA	106 (28.2)	45 (27.3)	61 (22.3)	0.95 (0.62-1.44)	0.7949	0.66 (0.45-0.97)	**0.0341**
	Rec.							
	GG/ GA	368 (97.9)	160 (97)	267 (97.4)	Ref.		Ref.	
	AA	8 (2.1)	5 (3)	7 (2.6)	1.06 (0.33-3.44)	0.9245	0.85 (0.29-2.50)	0.7646
	Overdom.							
	GG/ AA	278 (73.9)	125 (75.8)	220 (80.3)	Ref.		Ref.	
	GA	98 (26.1)	40 (24.2)	54 (19.7)	0.94 (0.61-1.44)	0.7644	0.66 (0.44-0.98)	**0.0378**

Analysis performed by multinomial logistic regression models adjusted for sex and ancestry. CO = controls; CA = cases; Ref. = reference; Codom. = codominant model; Dom. = dominant model; Rec. = recessive model; Overdom. = overdominant model.

**Table 2:** Association analysis by multinomial logistic regression models for associated SNPs in *CACNA2D3*, *IRGM* and *LRRK2* with leprosy clinical forms in Manaus (MAN) and Rondonópolis (ROO).

		Manaus^1^						
SNP	Model	CO (%)	PB (%)	MB (%)	OR (CI 95%)^PB^	p-value^PB^	OR (CI 95%)^MB^	p-value^MB^
rs1449325	Codom.							
	TT	324 (47.2)	47 (54.7)	87 (46.3)	Ref.		Ref.	
	TC	282 (41)	16 (18.6)	40 (21.3)	0.45 (0.25-0.81)	**0.0071**	0.55 (0.34-0.87)	**0.0114**
	CC	81 (11.8)	23 (26.7)	61 (32.4)	1.74 (0.98-3.06)	0.0567	2.42 (1.51-3.89)	**0.0003**
	Dom.							
	TT	324 (47.2)	47 (54.7)	87 (46.3)	Ref.		Ref.	
	TC/ CC	363 (52.8)	39 (45.3)	101 (53.7)	0.76 (0.48-1.20)	0.2456	1.01 (0.69-1.48)	0.9538
	Rec.							
	TT/ TC	606 (88.2)	63 (73.3)	127 (67.6)	Ref.		Ref.	
	CC	81 (11.8)	23 (26.7)	61 (32.4)	2.34 (1.37-4.01)	**0.0020**	3.07 (1.97-4.80)	**7.77E-07**
	Overdom.							
	TT/ CC	405 (59)	70 (81.4)	148 (78.7)	Ref.		Ref.	
	TC	282 (41)	16 (18.6)	40 (21.3)	0.39 (0.22-0.67)	**0.0008**	0.41 (0.27-0.64)	**8.45E-05**
rs1375515	Codom.							
	TT	245 (26.7)	40 (34.2)	58 (24.6)	Ref.		Ref.	
	TC	475 (51.7)	50 (42.7)	122 (51.7)	0.76 (0.46-1.25)	0.2732	0.92 (0.61-1.38)	0.6740
	CC	198 (21.6)	27 (23.1)	56 (23.7)	1.17 (0.69-1.99)	0.5675	0.83 (0.52-1.34)	0.4501
	Dom.							
	TT	245 (26.7)	40 (34.2)	58 (24.6)	Ref.		Ref.	
	TC/ CC	673 (73.3)	77 (65.8)	178 (75.4)	0.89 (0.56-1.42)	0.6338	0.89 (0.60-1.31)	0.5487
	Rec.							
	TT/ TC	720 (78.4)	90 (76.9)	180 (76.3)	Ref.		Ref.	
	CC	198 (21.6)	27 (23.1)	56 (23.7)	1.41 (0.93-2.15)	0.1037	0.89 (0.61-1.29)	0.5325
	Overdom.							
	TT/ CC	443 (48.3)	67 (57.3)	114 (48.3)	Ref.		Ref.	
	TC	475 (51.7)	50 (42.7)	122 (51.7)	0.69 (0.47-1.02)	0.0660	1.01 (0.73-1.40)	0.9606
rs13361189	Codom.							
	TT	624 (67)	87 (71.9)	172 (67.2)	Ref.		Ref.	
	TC	276 (29.6)	31 (25.6)	75 (29.3)	0.80 (0.52-1.23)	0.3001	0.90 (0.64-1.28)	0.5694
	CC	31 (3.3)	3 (2.5)	9 (3.5)	0.98 (0.37-2.61)	0.9618	0.75 (0.32-1.79)	0.5199
	Dom.							
	TT	624 (67)	87 (71.9)	172 (67.2)	Ref.		Ref.	
	TC/ CC	307 (33)	34 (28.1)	84 (32.8)	0.82 (0.54-1.23)	0.3303	0.89 (0.64-1.24)	0.4797
	Rec.							
	TT/ TC	900 (96.7)	118 (97.5)	247 (96.5)	Ref.		Ref.	
	CC	31 (3.3)	3 (2.5)	9 (3.5)	1.04 (0.39-2.76)	0.9329	0.78 (0.33-1.83)	0.5622
	Overdom.							
	TT/ CC	655 (70.4)	90 (74.4)	181 (70.7)	Ref.		Ref.	
	TC	276 (29.6)	31 (25.6)	75 (29.3)	0.80 (0.52-1.22)	0.2995	0.92 (0.65-1.29)	0.6237
rs11167514	Codom.							
	GG	594 (64.1)	83 (68)	160 (62.5)	Ref.		Ref.	
	GC	294 (31.7)	34 (27.9)	82 (32)	0.79 (0.52-1.20)	0.2747	0.95 (0.68-1.33)	0.7690
	CC	38 (4.1)	5 (4.1)	14 (5.5)	1.03 (0.42-2.53)	0.9529	1.21 (0.59-2.49)	0.6010
	Dom.							
	GG	594 (64.1)	83 (68)	160 (62.5)	Ref.		Ref.	
	GC/ CC	332 (35.9)	39 (32)	96 (37.5)	0.82 (0.55-1.22)	0.3263	0.98 (0.71-1.36)	0.9099
	Rec.							
	GG/ GC	888 (95.9)	117 (95.9)	242 (94.5)	Ref.		Ref.	
	CC	38 (4.1)	5 (4.1)	14 (5.5)	1.11 (0.45-2.70)	0.8244	1.23 (0.60-2.51)	0.5651
	Overdom.							
	GG/ CC	632 (68.3)	88 (72.1)	174 (68)	Ref.		Ref.	
	GC	294 (31.7)	34 (27.9)	82 (32)	0.79 (0.52-1.20)	0.2660	0.94 (0.67-1.31)	0.7053
rs7962370	Codom.							
	GG	684 (73.7)	96 (78.7)	185 (72.3)	Ref.		Ref.	
	GA	224 (24.1)	25 (20.5)	65 (25.4)	0.75 (0.47-1.20)	0.2339	1.01 (0.70-1.45)	0.9605
	AA	20 (2.2)	1 (0.8)	6 (2.3)	0.37 (0.05-2.81)	0.3346	1.44 (0.50-4.12)	0.4986
	Dom.							
	GG	684 (73.7)	96 (78.7)	185 (72.3)	Ref.		Ref.	
	GA/ AA	244 (26.3)	26 (21.3)	71 (27.7)	0.73 (0.46-1.15)	0.1686	1.04 (0.73-1.47)	0.8359
	Rec.							
	GG/ GA	908 (97.8)	121 (99.2)	250 (97.7)	Ref.		Ref.	
	AA	20 (2.2)	1 (0.8)	6 (2.3)	0.39 (0.05-2.99)	0.3654	1.43 (0.50-4.09)	0.4997
	Overdom.							
	GG/ AA	704 (75.9)	97 (79.5)	191 (74.6)	Ref.		Ref.	
	GA	224 (24.1)	25 (20.5)	65 (25.4)	0.77 (0.48-1.22)	0.2619	1.00 (0.70-1.43)	0.9955
		**Rondonópolis** ^**2**^						
**SNP**	**Model**	**CO (%)**	**PB (%)**	**MB (%)**	**OR (CI 95%)** ^**PB**^	**p-value** ^**PB**^	**OR (CI 95%)** ^**MB**^	**p-value** ^**MB**^
rs1449325	Codom.							
	TT	107 (33.2)	25 (31.6)	88 (36.4)	Ref.		Ref.	
	TC	143 (44.4)	30 (38)	82 (33.9)	0.91 (0.51-1.63)	0.7611	0.70 (0.47-1.03)	0.0702
	CC	72 (22.4)	24 (30.4)	72 (29.8)	1.44 (0.77-2.69)	0.2546	1.24 (0.81-1.89)	0.3328
	Dom.							
	TT	107 (33.2)	25 (31.6)	88 (36.4)	Ref.		Ref.	
	TC/ CC	215 (66.8)	54 (68.4)	154 (63.6)	1.09 (0.65-1.83)	0.7448	0.89 (0.62-1.24)	0.4639
	Rec.							
	TT/ TC	250 (77.6)	55 (69.6)	170 (70.2)	Ref.		Ref.	
	CC	72 (22.4)	24 (30.4)	72 (29.8)	1.51 (0.88-2.59)	0.1330	1.49 (1.02-2.17)	**0.0386**
	Overdom.							
	TT/ CC	179 (55.6)	49 (62)	160 (66.1)	Ref.		Ref.	
	TC	143 (44.4)	30 (38)	82 (33.9)	0.78 (0.47-1.28)	0.3238	0.64 (0.45-0.90)	**0.0105**

Analysis performed by multinomial logistic regression models adjusted for: (1) sex, age and ancestry and (2) sex and age. CO = controls; CA = cases; Ref. = reference; Codom. = codominant model; Dom. = dominant model; Rec. = recessive model; Overdom. = overdominant model.

Furthermore, significant associations in the same direction as those detected in the binomial models with leprosy *per se* were also detected by multinomial models for rs1375515 in *CACNA2D3*, rs13361189 and rs11167514 in *IRGM* with PB and MB forms in RIO, which was not replicated in MAN (Table 1). So, these were consequently not tested in ROO. After FDR correction (Table G in S1 Text), considering the overdominant model for rs1375515, only the association with clinical form MB in RIO remained statistically significant (FDR_p = 0.0341). For rs13361189 and rs11167514, for which the recessive model was considered, the associations with clinical form MB were also maintained after FDR correction (FDR_p = 0.0341 for both SNPs). The adjusted p-values for the association with these three SNPs with PB form in RIO were close to the FDR significance threshold but did not remain statistically significant.

A protection association of A carriers genotypes of the SNP rs7962370 in *LRRK2* with MB form in the dominant model (ORdomA.car = 0.66; p = 0.0341) in RIO was exclusively detected by multinomial logistic regression. This was not replicated in MAN and consequently was not tested in ROO (Table 1). After FDR correction (Table G in S1 Text), considering the dominant model for rs7962370, the described association in RIO remained statistically significant (FDR_p = 0.0341).

### The haplotype C/G/G/T/C of rs7308720/rs7962370/rs10878434/rs3761863/rs7133914 in *LRRK2* has a protection association with leprosy *per se*

Association analyses were performed for different haplotype combinations involving SNPs of each candidate gene in RIO. For all comparison models, the most frequent haplotype in each population was adopted as reference. No significant association was detected for haplotype combinations of SNPs in *CACNA2D3* (Table H in S1 Text). We observed a risk association of the haplotype C/C of rs13361189/rs11167514 in *IRGM* with leprosy *per se* in RIO (ORhapC/C = 1.27; p = 0.0331), which corroborates the findings from the individual analyses for such SNPs (Table I in S1 Text).

In addition, a protection association of the haplotype C/G/G/T/G of rs7308720/rs7133914/rs10878434/rs3761863/rs7962370 in *LRRK2*, respectively, was observed with leprosy *per se* (OR=0.44; p = 0.0121). Importantly, the only SNP whose allele varies compared to the reference haplotype (C/G/G/C/G) is rs3761863, which appears to be driving this association. This result demonstrates the importance of haplotype analysis for multifactorial outcomes, as no significant associations were observed when SNPs were individually analyzed (Table 3).

**Table 3:** Association analysis for haplotype combinations of SNPs in *LRRK2* with *leprosy per se* in Rio de Janeiro (RIO).

rs7308720	rs7962370	rs10878434	rs3761863	rs7133914	Freq. CO.	Freq. CA.	OR (CI 95%)	p-value
C	G	G	C	G	0.47167	0.45004	Ref.	–
C	G	A	T	G	0.19516	0.20724	0.93 (0.66-1.31)	0.6741
C	A	G	T	G	0.12905	0.14228	0.80 (0.55-1.17)	0.2491
G	G	G	C	A	0.07414	0.08695	0.98 (0.62-1.56)	0.9332
**C**	**G**	**G**	**T**	**G**	0.05269	0.03414	0.44 (0.24-0.84)	**0.0121**

Analysis performed by logistic regression models in the populations adjusted by sex and ancestry. CO = controls; CA = cases; Ref. = reference; Freq. = frequency.

### Associated SNPs in *CACNA2D3* and *LRRK2* have an eQTL effect

Gene expression analyses were also performed to investigate the functional role of the associated SNPs at *CACNA2D3*, *IRGM*, and *LRRK2*. Genotype/phenotype correlations were investigated using blood samples and skin biopsies from patients with leprosy *per se* in addition to data retrieved from public databases.

**Fig 2:**
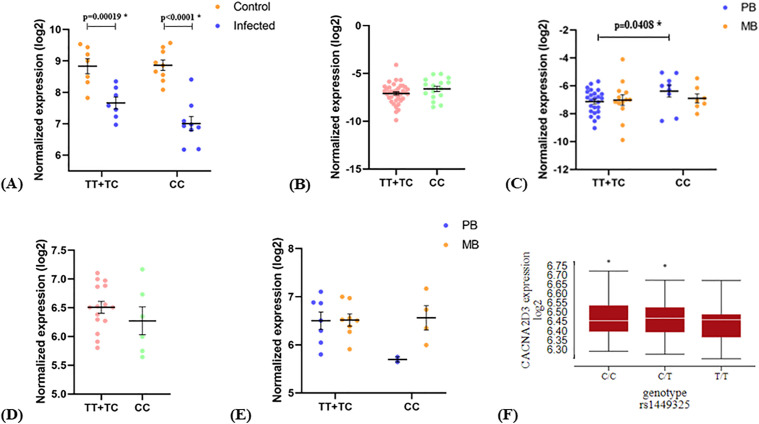
Expression analyses of *CACNA2D3* by rs1449325 genotypes. (A) macrophages against rs1449325 genotypes in the recessive model comparing controls and infected with *M. leprae*; (B) whole blood samples from leprosy *per se* patients against genotypes in the recessive model; (C) same samples as B, comparing PB (blue) and MB (orange) patients in the same model; (D) skin biopsies from leprosy *per se* patients against genotypes in the recessive model; (E) same samples as in D, comparing PB (blue) and MB (orange) patients in the same model; (F) eQTL analysis from public database data in monocytes stimulated with LPS for 6h. The means of the normalized expression values of each group of genotypes were compared using Paired T test or Welch’s T-test, when the data followed a normal distribution and Mann-Whitney test, when otherwise. * = significant difference between the expression levels of the compared groups; PB = paucibacillary; MB = multibacillary.

First, we confirmed that *M. leprae* in vitro infection in macrophage-derived monocytes showed a significant reduction of *CACNA2D3* expression independently of the genotype of rs1449325 (Fig 2A). We observed an eQTL effect of rs1449325 on *CACNA2D3* mRNA expression in CC whole blood samples compared to TT + TC samples from leprosy patients (Fig 2B and 2C), but not in skin samples (Figs 2D and 2E). Data from public databases [55] indicate that the T allele of rs1449325 is significantly associated with a lower expression of *CACNA2D3* in monocytes after LPS stimulation for 6h (Fig 2F). No eQTL effects associated with the genotypes of rs1375515 in *CACNA2D3* and SNPs in *IRGM* were observed (Figs G, H, I and J in S1 Text).

**Fig 3:**
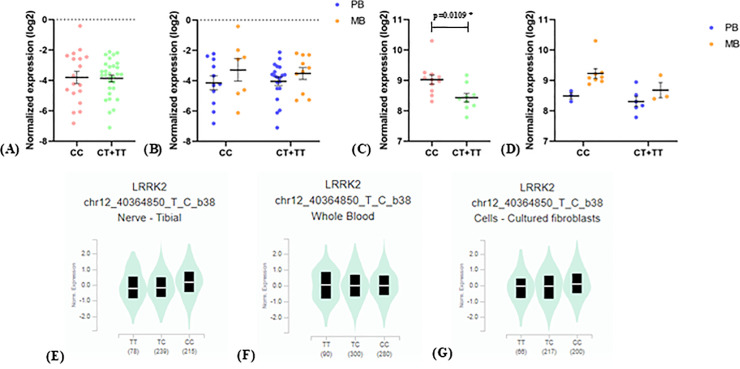
Expression analyses of *LRRK2* against rs3761863 genotypes. (A) whole blood samples from leprosy *per se* patients against their rs3761863 genotypes in the dominant model; (B) same samples as in A, comparing PB (blue) and MB (orange) patients; (C) skin biopsies from leprosy *per se* patients against their rs3761863 genotypes in the dominant model; (D) same samples as in C, comparing PB (blue) and MB (orange) patients. The means of the normalized expression values of each genotype group were compared using Welch’s t-test, when the data followed a normal distribution and Mann-Whitney test, when the data were nonparametric. * = significant difference between the expression levels of the compared groups; PB = paucibacillary; MB = multibacillary. eQTL analyses from public databases of rs3761863 for *LRRK2* in: (E) tibial nerve; (F) whole blood and; (G) cultured fibroblasts from healthy subjects.

We also observed a genotype-associated eQTL effect for rs3761863 in *LRRK2*, since there was a significant decrease in the expression of this gene in skin biopsies of patients with leprosy *per se* when comparing T-carriers genotypes to CC (Fig 3C), but not in whole blood samples (Fig 3A and 3B). This comparison model was adopted due to the protective association of the C/G/G/T/G haplotype of rs7308720/rs7133914/rs10878434/rs3761863/rs7962370 in *LRRK2* with leprosy *per se*. Data from public databases [53,54] corroborate this result, showing that the T allele is related to a lower expression of *LRRK2* in tibial nerve samples, whole blood and fibroblasts from healthy individuals (Fig 3E-3G). However, in monocytes unstimulated and stimulated with MDP for 6 hours an inverse effect is observed (Fig K in S1 Text).

## Discussion

Autophagy is involved in pathogen clearance as well as antigen presentation, which is a key step for CD4 + T-lymphocyte activation [12,17,18]. This process depends on the activity of a complex machinery regulated by different pathways in different steps, both at the protein and transcriptional level [19–21]. In this study, we observed that SNPs in *CACNA2D3*, *IRGM*, and *LRRK2* are associated with leprosy susceptibility and its clinical forms. Since these genes are involved, among other pathways, in autophagy regulation, it is reasonable to think that such polymorphisms are related to alterations in different points of this process.

*CACNA2D3* encodes the α2δ3 subunit of the voltage-gated calcium channel (Cav). This channel is mainly located on the plasma membrane and the endoplasmic reticulum (ER) membrane, where it directs intracellular Ca^2^^+^ influx and stores this cation in the ER, respectively [42,43]. We detected a peculiar association pattern for rs1449325 in *CACNA2D3*, in both overdominant and recessive models, where TC shows protection and CC a risk association with leprosy. This was consistently replicated in the three Brazilian populations tested, further emphasizing the importance of this result. *M. leprae* reduced *CACNA2D3* mRNA levels after in vitro infection of monocytes. Likewise, *M. tuberculosis* has developed a specific strategy to actively inhibit Ca^2^^+^ mediated signal transduction. This pathogen actively induces the expression of miRNA27a, which inhibits *CACNA2D3* expression, aiming to favor its survival [44].

We observed an eQTL effect of rs1449325 for *CACNA2D3* indicating that TT genotype is related to a lower expression of this gene after LPS stimulation. In different cancer models, *CACNA2D3* mRNA levels were directly correlated with Ca^2^^+^ levels [56,57]. Gene expression from whole blood indicated that CC individuals have higher *CACNA2D3* mRNA levels. Collectively, our genetic and functional data indicate that rs1449325 regulate levels of *CACNA2D3* mRNA, suggesting it might also influence intracellular Ca^2^^+^ levels. Data indicate increasing levels from TT < CT < CC. It suggests that low or high levels can be damaging and associated with risk, while moderate levels are protective for leprosy.

Ca^2^^+^ plays a dual role in autophagy regulation, exerting dose- and context-dependent effects, both promoting this process through AMPK activation by CAMKKβ in the cytoplasm and inhibiting it by stimulating ATP production when transferred from the ER to mitochondria in oscillatory patterns [58,59]. Although Ca^2^^+^ has a dual effect, it is reasonable to hypothesize that it contributes mostly to autophagy induction, as *M. leprae* probably has also developed strategies to actively inhibit calcium-mediated signaling. Cytosolic Ca^2^^+^ is also related to higher NO production, microbicidal granules, and pro-inflammatory mediators, which contribute to better control of infection [32]. Furthermore, it has been observed that in macrophages infected with mycobacteria (*M. marinum* and *M. tuberculosis*), excessive TNF production can trigger a form of programmed necrosis. The increase in TNF production due to infection stimulates ROS production in mitochondria, which triggers a signaling cascade that results in Ca^2^^+^ release from the ER to mitochondria through activation of ryanodine receptors (RYR). This results in mitochondrial Ca^2^^+^ overload, triggering necrosis in infected macrophages, compromising granuloma integrity, and promoting the spread of mycobacteria and their extracellular growth [60,61].

**Fig 4:**
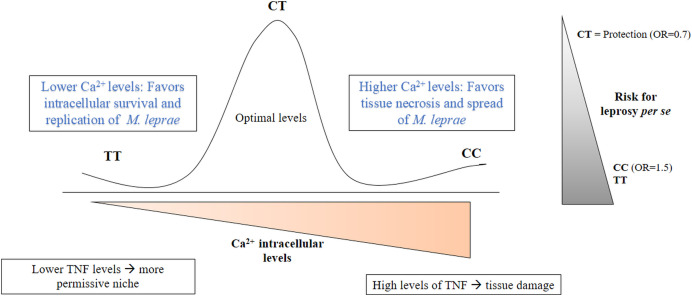
Probable functional effects related to the association of different genotypes of rs1449325 in *CACNA2D3* with leprosy *per se.* The TT genotype is related to a lower expression of this gene and, consequently, to lower levels of intracellular Ca^2^^+^, which leads to a negative balance of autophagy, making the environment more permissive and favoring the survival and replication of *M. leprae*. The CC genotype is related to a higher expression of *CACNA2D3* and, consequently, to higher levels of Ca^2^^+^, which is related to an upregulation of the necrosis process triggered by high levels of TNF in cells infected by mycobacteria. The CT genotype is related to intermediate levels of Ca^2^^+^, which would lead to a balance between the pathways that regulate autophagy and other key processes, favoring a more efficient cellular immune response and, therefore, conferring protection for leprosy.

The protective association observed for CT genotype suggests that intermediate intracellular Ca^2^^+^ levels may favor a balanced regulation of autophagy and other calcium-dependent processes, resulting in a more favorable scenario for the infection control (Fig 4). In tuberculosis, verapamil, valproic acid among several other medications inhibit eukaryotic Ca^2^^+^ efflux, which is associated with more effective anti-TB treatments since higher concentrations of anti-TB drugs are reached inside Mtb-infected host cells [62,63]. Thus, it is likely that host-based adjuvanted therapies for high producers, in this case, CC individuals, Ca^2^^+^ inhibitors could have benefits reducing the levels of calcium that would lead to faster, optimized and personalized treatment for leprosy. Autophagy has been evaluated as a target for drug development and repurposing against cutaneous mycobacterial infection [64]. Initial studies assessing the impact of verapamil and its analogs on macrophages infected with *M. tuberculosis* demonstrated that the structural analog KSV21 enhanced the inhibitory antimicrobial activity of Isoniazid and Rifampicin in an additive manner [65].

LRRK2 is a multi-domain protein primarily located on cell membranes, regulating processes like autophagy, often in interaction with cytoplasmic Ca^2^^+^ [20]. Both LRRK2 and Ca^2^^+^ play dual roles in autophagy regulation, acting as activators or inhibitors depending on the cellular context. LRRK2 interacts with CD38 on plasma membrane, activating pathways that release the Ca^2^^+^ stored inside the lysosomes and increase cytoplasmic Ca^2^^+^ levels. This results in the activation and translocation to the nucleus of the transcription factor TFEB, which positively regulates the expression of key genes involved in autophagy [21,55,66,67]. However, the release of Ca^2^^+^ from lysosomes can impair the fusion of autophagosomes with them. Therefore, LRRK2 activity contributes to autophagosomes accumulation in the cytoplasm, since it induces the initial activation of autophagy, but impairs the autophagic flux and, consequently, the pathogen clearance [22,56].

In Rio de Janeiro, A carriers genotypes of rs7962370, located between the *LRRK2* and *MUC19* genes, was associated with protection against MB leprosy. This SNP has also been associated with Crohn’s disease [57]. It was observed that the A allele of this SNP is related to a decrease in LRRK2 expression in monocytes after LPS stimulation. Therefore, the protective effect of this SNP could be related to greater efficiency in *M. leprae* clearance. However, differences in genotype frequencies between Rio de Janeiro and Manaus suggest that genetic background may explain the lack of replication of this finding in Manaus.

NFAT (nuclear factor of activated T cells) is associated with an increase in the expression of genes encoding cytokines and other key proteins involved in the inflammatory response regulation. The increase in cytosolic Ca^2^^+^ induces the translocation of NFAT to the nucleus [66]. However, LRRK2 retains these molecules in the cytoplasm through a complex mechanism mediated by Ca^2^^+^, negatively regulating these genes expression. There is evidence that NFAT acts in the innate immune response regulation in macrophages, dendritic cells and neutrophils, in addition to being involved in T cell cytokines production, neuronal differentiation, stem cell maintenance and cardiac development [68–70].

rs3761863, located in the WD40 domain of *LRRK2*, is a nonsynonymous exonic variant that leads to the substitution of methionine (T allele) with threonine (C allele) at position 2397. The T allele has a risk association with Crohn’s disease, which is related to a decrease in LRRK2 cellular levels [69]. In Han Chineses, the TT genotype confers risk for leprosy *per se* [71]. A study in Vietnam showed that the amino acid methionine in this position is related to a decrease in LRRK2 half-life, decreasing the levels of NFAT retained in the cytosol, inducing its translocation to the nucleus [72]. We observed that the T allele of rs3761863 drives the protection association of the haplotype C/G/G/T/G of rs7308720/rs7133914/ rs10878434/rs3761863/rs7962370 with leprosy *per se* in Rio de Janeiro. Furthermore, we observed that this allele is related to a lower expression of LRRK2 in skin biopsy samples from leprosy patients, as well as in tibial nerve and fibroblast samples of healthy individuals. A decrease in both expression and half-life of LRRK2 leads to greater translocation of NFAT to the nucleus, which contributes to control of *M.leprae* infection.

Differences in linkage disequilibrium (LD) patterns between Brazilian and other populations highlight the need to consider haplotypes for better understanding the relationship between genetic variations and leprosy. Association analysis considering haplotype combinations becomes even more relevant in this scenario, where the genetic landscape can directly influence the functional effect related to one or more polymorphisms. However, it was recently demonstrated that *LRRK2* knockout in RAW cells resulted in reduced ROS production and reduced NF-Kb pathway activation in response to BCG and *M. leprae* infection. Diminished expression of MCP-1, TNF and IL-6 in cell cultures was also observed in these settings [73]. These data corroborate the idea that LRRK2 is a protein that acts in different pathways and that its expression levels can influence leprosy outcome, both in terms of controlling and favoring the infection. Therefore, it is clear that the role of *LRRK2* in leprosy outcome still requires further studies until it is fully elucidated.

*IRGM* encodes the Immunity-related GTPase family M protein (IRGM), which positively regulates autophagy at different steps [74,75]. Its expression can be strongly induced in monocytes by infection with bacteria associated with Crohn’s disease or by treatment with microbial components, such as LPS and MDP. Furthermore, IRGM interacts with NOD2, promoting its oligomerization, which appears to be essential for its biochemical functions and properties. In this sense, an increased IRGM expression could confer protection against infectious bacterial diseases [24–27]. We observed a risk association of the CC genotypes of rs13361189 and rs11167514 with leprosy *per se* in Rio de Janeiro, which was not replicated in Manaus. These SNPs are in moderate LD in Rio de Janeiro and in strong LD in Manaus. Therefore, it is likely that the association observed for rs11167514 in Rio de Janeiro may be driven by rs13361189, due to its functional role already reported in the literature.

In Han Chineses, a risk association of the C carriers genotypes of rs13361189 with leprosy was also observed. Furthermore, it was also seen that PBMCs from CC individuals, when infected with *M. leprae*, express significantly higher levels of INF-γ and IL-4 [76]. Previous studies have shown a strong association of *IRGM* polymorphisms with Crohn’s disease and tuberculosis in different populations [77–80]. rs13361189 is located in *IRGM* promoter and data from RNA-Seq showed a decreased expression of this in whole blood from C carriers patients with Crohn’s disease. Furthermore, alterations in the expression levels of TNF and other genes involved with autophagy were also detected for these patients [81]. In this scenario, the IRGM decreased expression results in autophagy downregulation and seems to be related to changes in the levels of important cytokines, which favors *M. leprae* survival and corroborates the obtained results. Fig L in S1 Text schematizes possible functional effects of genetic variations in *LRRK2* and *IRGM*.

In this work, we described unprecedented results showing the association of *CACNA2D3* with leprosy, in addition to the new association of SNPs in *LRRK2* and *IRGM* in the Brazilian population. The understanding of results from association studies involving Brazilians becomes complex, because they make up a highly admixed population. Although further studies are still needed to understand the exact biological role of these associated SNPs, the results provide additional evidence supporting the involvement of *CACNA2D3*, *IRGM*, and *LRRK2* in the immunogenetic landscape of leprosy. Overall, our findings contribute to a greater understanding of how regulatory pathways of different processes contribute to determining leprosy outcome.

### Study limitations

In this study, we did not perform analyses to distinguish whether the leprosy cases were caused by *M. leprae* or *M. lepromatosis*. Diagnosis was based on standard criteria, which do not allow species-level discrimination. In Brazil, *M. lepromatosis* cases are rare, likely underdiagnosed, and not routinely investigated. Given the close genetic relationship between these species and their shared host–pathogen interaction mechanisms, this limitation is unlikely to substantially impact the host genetic associations observed [82].

Although the association analyses performed in this study present strengths, including a reasonable sample size and cohorts from different regions of Brazil with distinct ancestry patterns and environmental exposures, some limitations should be acknowledged. First, healthy controls were not matched to cases, resulting in differences between groups for relevant variables such as sex, age, and ancestry. To minimize the impact of these differences and to increase the reliability of the results, all statistical association analyses were adjusted for these confounding variables. Nevertheless, the environmental and socioeconomic conditions to which healthy controls are exposed may not fully reflect those of leprosy cases, given the strong association of the disease with poverty and prolonged exposure to infected individuals. However, recruiting controls under comparable conditions could likely hinder the attainment of such a considerable sample size and reduce the statistical power, which are essential for robust genetic association studies.

Second, we acknowledge that stratifying the case cohort into the PB and MB clinical forms results reduces statistical power compared to leprosy *per se* analyses. However, leprosy is a disease characterized by wide phenotypic heterogeneity, making this approach particularly relevant for elucidating not only of disease susceptibility but also the genetic determinants of distinct clinical forms among affected individuals.

Third, we saw that associations found for several SNPs in Rio de Janeiro were not replicated in Manaus, which may be related to the considerable difference in genetic background of these populations. Replication of genetic associations across independent populations strengthens the evidence that a given SNP is truly associated with leprosy susceptibility and may indicate a potential causal role. However, the absence of replication does not necessarily preclude biological relevance in leprosy. Instead, such discrepancies may reflect differences in linkage disequilibrium (LD) structure across populations, whereby the tested SNPs tag the causal variant in one population but not in another [83].

Furthermore, leprosy is a complex disease characterized by heterogeneous clinical outcomes, in which multiple genetic variants contribute modest effects to disease susceptibility and immune response modulation. Genes such as *IRGM* and *LRRK2*, which play central roles in autophagy and innate immune signaling, are likely influenced by broader genetic contexts that vary among populations. Thus, variations in LD patterns, as well as in the frequencies of various markers across the genome, contribute to shaping different “genetic landscapes” in different populations. This also influences association results, since other markers across the genome can exert “compensatory” or “enhancing” effects, modulating the functional impact of individual variants and contributing to the heterogeneity of association results observed between cohorts [84].

Fourth and last, to investigate the biological plausibility of the identified associations, functional analyses were performed to explore whether the associations found could be explained by possible eQTL effects on the genes where they were located. Although regulatory effects on gene expression may explain some of the observed associations, it is important to note that the causal relationship between an associated SNP and the pathogenesis of leprosy will not necessarily be explained solely by effects at the transcriptional level. Some variants may also have some impact on the activity, stability, or synthesis of the encoded protein, which would require further studies to be evaluated [85–87].

## Supporting information

S1 Text Table A: Demographic characteristics of the populations studied. Table B: Information on the selected candidate SNPs located in the regions of *CACNA2D3*, *IRGM* and *LRRK2.* Table C: Demographic characteristics of the samples involved in the functional assays. Table D: Description of primers used in gene expression analysis. Table E: Association analysis by logistic regression models for associated SNPs in *CACNA2D3*, *IRGM* and *LRRK2* with leprosy *per se* in Rio de Janeiro (RIO), Manaus (MAN) and Rondonópolis (ROO). Table F: Association analysis by logistic regression models for non-associated SNPs in *CACNA2D3* and *LRRK2* with leprosy *per se* in Rio de Janeiro (RIO). Table G: Nominal p-values and false discovery rate (FDR)-adjusted p-values for SNP association analyses in populations of Rio de Janeiro (RIO) and Manaus (MAN). Table H: Association analysis for haplotype combinations of SNPs in *CACNA2D3* with leprosy *per se* in Rio de Janeiro (RIO). Table I: Association analysis for haplotype combinations of SNPs in *IRGM* with leprosy *per se* in Rio de Janeiro (RIO). Fig A: Description of the SNPs selection methodology. Fig B: Principal Component Analysis (PCA) of the SNPs located from a region of 5,000 bp upstream and downstream of each candidate gene in the parental populations of the 1000 Genomes Project: *CACNA2D3* (A); *IRGM* (B) and; *LRRK2* (C). Fig CLinkage disequilibrium map between pre-selected SNPs in *CACNA2D3* region from annotation, PCA and frequency analysis for African (A) and European (B) parental populations of the 1000 Genomes Project. Fig D: Linkage disequilibrium map between pre-selected SNPs in *IRGM* region from annotation, PCA and frequency analysis for African (A) and European (B) parental populations of the 1000 Genomes Project. Fig E: Linkage disequilibrium map between pre-selected SNPs in *LRRK2* region from annotation, PCA and frequency analysis for African (A) and European (B) parental populations of the 1000 Genomes Project. Fig F: LD maps between selected SNPs in different genes for Rio de Janeiro (RIO) control cohort. Fig G: Expression analyses of *CACNA2D3* against rs1449325 genotypes in different models. Fig H: Expression analyses of *CACNA2D3* against rs1375515 genotypes. Fig I: eQTL analysis from public database data of rs1375515 for *CACNA2D3* in monocytes stimulated with LPS for 6h. Fig J: Expression analyses of *IRGM* against genotypes of associated SNPs. Fig K: eQTL analyses from public databases of rs3761863 for *LRRK2* in: (A) unstimulated monocytes and; stimulated with (B) MDP for 6h. Fig L: Possible impacts of variants in the *LRRK2* and *IRGM* in different scenarios of *M. leprae* infection in macrophages.
(DOCX)

S1 Checklist STROBE checklist for case-control studies used to guide the reporting of this study. Information on the STROBE Initiative is available at http://www.strobe-statement.org
(DOCX)
