# Supplementary material for: Functional single nucleotide polymorphisms in CACNA2D3 and other autophagy-related genes are associated with leprosy among Brazilians
Source: PLoS Negl Trop Dis. 2026 Apr 27;20(4):e0014241. doi: 10.1371/journal.pntd.0014241 (PMC13120706; doi:10.1371/journal.pntd.0014241)

**SUPPORTING INFORMATION**

**TABLES**

**Table A: Demographic characteristics of the populations studied.**

|  |  | **Rio de Janeiro** | | **Manaus** | | **Rondonópolis** | |
| --- | --- | --- | --- | --- | --- | --- | --- |
| **Cohort** | | **CO** | **CA** | **CO** | **CA** | **CO** | **CA** |
| **N** | | 579 | 759 | 967 | 407 | 357 | 411 |
| **Gender (%)** | **M** | 320 (55.3) | 495 (65.2) | 526 (54.4) | 280 (68.8) | 217 (60.8) | 250 (60.8) |
|  | **F** | 259 (44.7) | 264 (34.8) | 441 (45.6) | 127 (31.2) | 140 (39.2) | 161 (39.2) |
| **Age (Mean)** | | 33.66 | 41.48 | 20.76 | 35.2 | 41.99 | 42.02 |
| **Ancestry (Mean %)** | **AFR** | 18.31 | 29.76 | 23.12 | 22.39 | 24.19 | 27.27 |
|  | **AMR** | 10.7 | 14.29 | 39.38 | 37.63 | 17.19 | 14.84 |
|  | **EUR** | 70.98 | 55.95 | 37.5 | 39.99 | 58.6 | 57.89 |
| **Clinical form (%)** | **PB** | - | 267 (35.2) | - | 130 (31.9) | - | 96 (23.4) |
|  | **MB** | - | 398 (64.8) | - | 277 (68.1) | - | 310 (76.6) |

Percentages of African (AFR), European (EUR) and Native-American (AMR) ancestries was calculated by a set of 46-AIMs-Indels to adjust for the genetic association studies, as described by Manta *et al* (2013). CO = Controls; CA = Cases.

**Table B: Information on the selected candidate SNPs located in the regions of *CACNA2D3*, *IRGM* and *LRRK2.***

| **SNP** | **Gene** | **Chromosome** | **Position** | **Alleles** | **Classification** | **eQTL effect**  **(GTEx portal) *** |
| --- | --- | --- | --- | --- | --- | --- |
| rs1449325 | *CANA2D3* | 3 | 54394366 | T > C | Intronic | - |
| rs1375515 | *CANA2D3* | 3 | 54476640 | C > T | Intronic | Nerve – Tibial |
| rs622326 | *CANA2D3* | 3 | 54677875 | T > C | Intronic | - |
| rs3732748 | *CANA2D3* | 3 | 54798258 | G > C | Exonic non-synonymous | Skin |
| rs13361189 | *IRGM* | 5 | 150223387 | T > C | Intergenic *SMIM3*/*IRGM* | - |
| rs11167514 | *IRGM* | 5 | 150230977 | G > C | Intergenic *IRGM*/*ZNF300* | - |
| rs7308720 | *LRRK2* | 12 | 40657700 | C > G | Exonic non-synonymous | Fibroblasts and skin |
| rs7133914 | *LRRK2* | 12 | 40702911 | G > A | Exonic non-synonymous | Fibroblasts and skin |
| rs10878434 | *LRRK2* | 12 | 40758291 | G > A | Intronic | Fibroblasts, nerve, etc |
| rs3761863 | *LRRK2* | 12 | 40758652 | C > T | Exonic non-synonymous | Whole blood, fibroblasts, nerve, etc. |
| rs7962370 | *LRRK2* | 12 | 40765929 | G > A | Intergenic *LRRK2*/*MUC19* | - |

*The respective SNPs have a significant eQTL effect for samples of the tissues described.

**Table C: Demographic characteristics of the samples involved in the functional assays.**

| **Assay / sample** | | **RNAseq skin biopsies** | **qPCR whole blood expression** |
| --- | --- | --- | --- |
| **N** | | 21 | 55 |
| **Clinical form (%)** | **PB** | 9 (42.9) | 34 (61.8) |
|  | **MB** | 12 (57.1) | 21 (38.2) |
| **Gender (%)** | **M** | 16 (76.2) | 33 (60) |
|  | **F** | 5 (23.8) | 22 (40) |
| **Age (mean)** | | 51 | 52 |

Table D: Description of primers used in gene expression analyses.

| **Gene** | **Prmer sequence (5’ – 3’)** | **Size (nt)** | **Tm (^o^C)** | **GC content** | **Amplicon (nt)** |
| --- | --- | --- | --- | --- | --- |
| ***LRRK2*** | F: ACAGCACAGCTAGGAAGC | 18 | 59,92 | 55,56% | 120 |
|  | R: ATTGATGTCCCAAACGGTCA | 20 | 59,94 | 45,00% |  |
| ***CACNA2D3*** | F: TAGAGCCAACAAGGAAAGCA | 20 | 59,6 | 45,00% | 111 |
|  | R: TCCACCAGGAACAAGACAAG | 20 | 60,07 | 50,00% |  |
| ***IRGM*** | F: AGTCTTAATGGAAGCTCGGG | 20 | 59,69 | 50,00% | 113 |
|  | R: GCACTGGAGTAAGCTCATCA | 20 | 59,91 | 50,00% |  |
| ***RPL13*** | F: GACAAGAAAAAGCGGATGGT | 20 | 56,63 | 45,00% | 111 |
|  | R: GTACTTCCAGCCAACCTCGT | 20 | 59,68 | 55,00% |  |
| ***RPS16*** | F: GCGCACGCTACAGTACAAG | 19 | 59,30 | 57,89% | 134 |
|  | R: AGATGGACTGACGGATAGCATA | 22 | 58,23 | 45,45% |  |

F= forward; R= reverse; nt = nucleotides.

**Table E: Association analysis by logistic regression models for associated SNPs in *CACNA2D3*, *IRGM* and *LRRK2* with leprosy *per se* in Rio de Janeiro (RIO), Manaus (MAN) and Rondonópolis (ROO).** Analysis performed using logistic regression models adjusted for: (1) sex and ancestry; (2) sex, age and ancestry; (3) sex, age and ancestry and; (4) sex and age. CO = controls; CA = cases; Ref. = reference; Codom. = codominant model; Dom. = dominant model; Rec. = recessive model; Overdom. = overdominant model; log Add = log. Additive model.

|  |  | **Rio de Janeiro^1^** | | | | **Rio de Janeiro^2^** | | | | **Manaus^3^** | | | | **Rondonópolis^4^** | | | |
| --- | --- | --- | --- | --- | --- | --- | --- | --- | --- | --- | --- | --- | --- | --- | --- | --- | --- |
| **SNP** | **Model** | **CO (%)** | **CA (%)** | **OR (CI 95%)^1^** | **p-value^1^** | **CO (%)** | **CA (%)** | **OR (CI 95%)^2^** | **p-value^2^** | **CO (%)** | **CA (%)** | **OR (CI 95%)^3^** | **p-value^3^** | **CO (%)** | **CA (%)** | **OR (CI 95%)^4^** | **p-value^4^** |
| rs1449325 | Codom. |  |  |  |  |  |  |  |  |  |  |  |  |  |  |  |  |
|  | TT | 138 (25.5) | 205 (31.8) | Ref. | **0.00566** | 73 (24.4) | 193 (31.5) | Ref. | **0.02636** | 324 (47.2) | 134 (48.9) | Ref. | **9.04E-10** | 107 (33.2) | 114 (35) | Ref. | **0.0237** |
|  | TC | 276 (50.9) | 272 (42.2) | 0.79 (0.59-1.07) |  | 156 (52.2) | 259 (42.3) | 0.71 (0.48-1.06) |  | 282 (41) | 56 (20.4) | 0.47 (0.31-0.7) |  | 143 (44.4) | 114 (35) | 0.75 (0.52-1.08) |  |
|  | CC | 128 (23.6) | 167 (25.9) | 1.3 (0.92-1.83) |  | 70 (23.4) | 160 (26.1) | 1.21 (0.76-1.92) |  | 81 (11.8) | 84 (30.7) | 2.28 (1.47-3.54) |  | 72 (22.4) | 98 (30.1) | 1.29 (0.86-1.94) |  |
|  | Dom. |  |  |  |  |  |  |  |  |  |  |  |  |  |  |  |  |
|  | TT | 138 (25.5) | 205 (31.8) | Ref. | 0.65472 | 73 (24.4) | 193 (31.5) | Ref. | 0.42039 | 324 (47.2) | 134 (48.9) | Ref. | 0.45148 | 107 (33.2) | 114 (35) | Ref. | 0.6637 |
|  | TC / CC | 404 (74.5) | 439 (68.2) | 0.94 (0.71-1.24) |  | 226 (75.6) | 419 (68.5) | 0.86 (0.59-1.25) |  | 363 (52.8) | 140 (51.1) | 0.88 (0.63-1.23) |  | 215 (66.8) | 212 (65) | 0.93 (0.67-1.29) |  |
|  | Rec. |  |  |  |  |  |  |  |  |  |  |  |  |  |  |  |  |
|  | TT / TC | 414 (76.4) | 477 (74.1) | Ref. | **0.00476** | 229 (76.6) | 452 (73.9) | Ref. | **0.03341** | 606 (88.2) | 190 (69.3) | Ref. | **1.44E-07** | 250 (77.6) | 228 (69.9) | Ref. | **0.0240** |
|  | CC | 128 (23.6) | 167 (25.9) | 1.51 (1.13-2.01) |  | 70 (23.4) | 160 (26.1) | 1.51 (1.03-2.21) |  | 81 (11.8) | 84 (30.7) | 3.06 (2.02-4.63) |  | 72 (22.4) | 98 (30.1) | 1.50 (1.05-2.15) |  |
|  | Overdom. |  |  |  |  |  |  |  |  |  |  |  |  |  |  |  |  |
|  | TT / CC | 266 (49.1) | 372 (57.8) | Ref. | **0.00443** | 143 (47.8) | 353 (57.7) | Ref. | **0.01012** | 405 (59) | 218 (79.6) | Ref. | **1.19E-07** | 179 (55.6) | 212 (65) | Ref. | **0.0145** |
|  | TC | 276 (50.9) | 272 (42.2) | 0.70 (0.55-0.9) |  | 156 (52.2) | 259 (42.3) | 0.65 (0.47-0.9) |  | 282 (41) | 56 (20.4) | 0.37 (0.25-0.54) |  | 143 (44.4) | 114 (35) | 0.67 (0.49-0.93) |  |
|  | log Add. |  |  |  |  |  |  |  |  |  |  |  |  |  |  |  |  |
|  | X 0.1.2 | 542 (45.7) | 644 (54.3) | 1.13 (0.95-1.34) | 0.15704 | 299 (32.8) | 612 (67.2) | 1.1 (0.87-1.37) | 0.42671 | 687 (71.5) | 274 (28.5) | 1.28 (1.03-1.6) | **0.02537** | 322 (49.7) | 326 (50.3) | 1.11 (0.91-1.36) | 0.3091 |
| rs1375515 | Codom. |  |  |  |  |  |  |  |  |  |  |  |  |  |  |  |  |
|  | TT | 173 (31.1) | 218 (32.6) | Ref. | **0.01785** | 92 (30.2) | 206 (32.4) | Ref. | **0.03696** | 245 (26.7) | 98 (27.8) | Ref. | 0.58552 |  |  |  |  |
|  | TC | 286 (51.3) | 300 (44.8) | 0.67 (0.51-0.89) |  | 162 (53.1) | 286 (45) | 0.63 (0.44-0.91) |  | 475 (51.7) | 172 (48.7) | 0.86 (0.62-1.2) |  |  |  |  |  |
|  | CC | 98 (17.6) | 151 (22.6) | 0.84 (0.59-1.19) |  | 51 (16.7) | 143 (22.5) | 0.88 (0.54-1.41) |  | 198 (21.6) | 83 (23.5) | 0.99 (0.67-1.48) |  |  |  |  |  |
|  | Dom. |  |  |  |  |  |  |  |  |  |  |  |  |  |  |  |  |
|  | TT | 173 (31.1) | 218 (32.6) | Ref. | **0.01206** | 92 (30.2) | 206 (32.4) | Ref. | **0.03550** | 245 (26.7) | 98 (27.8) | Ref. | 0.50399 |  |  |  |  |
|  | TC / CC | 384 (68.9) | 451 (67.4) | 0.72 (0.55-0.93) |  | 213 (69.8) | 429 (67.6) | 0.69 (0.48-0.98) |  | 673 (73.3) | 255 (72.2) | 0.90 (0.66-1.23) |  |  |  |  |  |
|  | Rec. |  |  |  |  |  |  |  |  |  |  |  |  |  |  |  |  |
|  | TT / TC | 459 (82.4) | 518 (77.4) | Ref. | 0.67471 | 254 (83.3) | 492 (77.5) | Ref. | 0.47402 | 720 (78.4) | 270 (76.5) | Ref. | 0.59868 |  |  |  |  |
|  | CC | 98 (17.6) | 151 (22.6) | 1.07 (0.79-1.45) |  | 51 (16.7) | 143 (22.5) | 1.16 (0.77-1.76) |  | 198 (21.6) | 83 (23.5) | 1.1 (0.78-1.54) |  |  |  |  |  |
|  | Overdom. |  |  |  |  |  |  |  |  |  |  |  |  |  |  |  |  |
|  | TT / CC | 271 (48.7) | 369 (55.2) | Ref. | **0.00770** | 143 (46.9) | 349 (55) | Ref. | **0.01210** | 443 (48.3) | 181 (51.3) | Ref. | 0.30106 |  |  |  |  |
|  | TC | 286 (51.3) | 300 (44.8) | 0.72 (0.56-0.92) |  | 162 (53.1) | 286 (45) | 0.66 (0.48-0.91) |  | 475 (51.7) | 172 (48.7) | 0.86 (0.65-1.14) |  |  |  |  |  |
|  | log Add. |  |  |  |  |  |  |  |  |  |  |  |  |  |  |  |  |
|  | X 0.1.2 | 557 (45.4) | 669 (54.6) | 0.88 (0.74-1.05) | 0.15340 | 305 (32.4) | 635 (67.6) | 0.89 (0.71-1.12) | 0.31870 | 918 (72.2) | 353 (27.8) | 0.99 (0.81-1.21) | 0.90833 |  |  |  |  |
| rs13361189 | Codom. |  |  |  |  |  |  |  |  |  |  |  |  |  |  |  |  |
|  | TT | 372 (66.4) | 395 (56.3) | Ref. | **0.04615** | 200 (65.4) | 375 (56.3) | Ref. | 0.20481 | 624 (67) | 259 (68.7) | Ref. | 0.65831 |  |  |  |  |
|  | TC | 174 (31.1) | 259 (36.9) | 1.13 (0.87-1.46) |  | 98 (32) | 249 (37.4) | 1.15 (0.82-1.63) |  | 276 (29.6) | 106 (28.1) | 0.87 (0.64-1.18) |  |  |  |  |  |
|  | CC | 14 (2.5) | 48 (6.8) | 2.16 (1.13-4.14) |  | 8 (2.6) | 42 (6.3) | 2.07 (0.86-4.97) |  | 31 (3.3) | 12 (3.2) | 1.02 (0.48-2.18) |  |  |  |  |  |
|  | Dom. |  |  |  |  |  |  |  |  |  |  |  |  |  |  |  |  |
|  | TT | 372 (66.4) | 395 (56.3) | Ref. | 0.13771 | 200 (65.4) | 375 (56.3) | Ref. | 0.23237 | 624 (67) | 259 (68.7) | Ref. | 0.40938 |  |  |  |  |
|  | TC / CC | 188 (33.6) | 307 (43.7) | 1.21 (0.94-1.55) |  | 106 (34.6) | 291 (43.7) | 1.23 (0.88-1.71) |  | 307 (33) | 118 (31.3) | 0.88 (0.66-1.19) |  |  |  |  |  |
|  | Rec. |  |  |  |  |  |  |  |  |  |  |  |  |  |  |  |  |
|  | TT / TC | 546 (97.5) | 654 (93.2) | Ref. | **0.02133** | 298 (97.4) | 624 (93.7) | Ref. | 0.11310 | 900 (96.7) | 365 (96.8) | Ref. | 0.87473 |  |  |  |  |
|  | CC | 14 (2.5) | 48 (6.8) | 2.06 (1.09-3.93) |  | 8 (2.6) | 42 (6.3) | 1.96 (0.82-4.67) |  | 31 (3.3) | 12 (3.2) | 1.06 (0.5-2.26) |  |  |  |  |  |
|  | Overdom. |  |  |  |  |  |  |  |  |  |  |  |  |  |  |  |  |
|  | TT / CC | 386 (68.9) | 443 (63.1) | Ref. | 0.59561 | 208 (68) | 417 (62.6) | Ref. | 0.58449 | 655 (70.4) | 271 (71.9) | Ref. | 0.36116 |  |  |  |  |
|  | TC | 174 (31.1) | 259 (36.9) | 1.07 (0.83-1.38) |  | 98 (32) | 249 (37.4) | 1.1 (0.78-1.55) |  | 276 (29.6) | 106 (28.1) | 0.87 (0.64-1.18) |  |  |  |  |  |
|  | log Add. |  |  |  |  |  |  |  |  |  |  |  |  |  |  |  |  |
|  | X 0.1.2 | 560 (44.4) | 702 (55.6) | 1.25 (1.01-1.55) | **0.03863** | 306 (31.5) | 666 (68.5) | 1.25 (0.94-1.67) | 0.11714 | 931 (71.2) | 377 (28.8) | 0.92 (0.71-1.18) | 0.51077 |  |  |  |  |
| rs11167514 | Codom. |  |  |  |  |  |  |  |  |  |  |  |  |  |  |  |  |
|  | GG | 327 (60.2) | 372 (53.1) | Ref. | **0.04923** | 176 (60.1) | 353 (53.1) | Ref. | 0.28851 | 594 (64.1) | 243 (64.3) | Ref. | 0.38078 |  |  |  |  |
|  | GC | 197 (36.3) | 270 (38.5) | 0.99 (0.77-1.28) |  | 107 (36.5) | 259 (38.9) | 1 (0.72-1.41) |  | 294 (31.7) | 116 (30.7) | 0.88 (0.65-1.2) |  |  |  |  |  |
|  | CC | 19 (3.5) | 59 (8.4) | 1.95 (1.11-3.44) |  | 10 (3.4) | 53 (8) | 1.81 (0.83-3.93) |  | 38 (4.1) | 19 (5) | 1.4 (0.73-2.67) |  |  |  |  |  |
|  | Dom. |  |  |  |  |  |  |  |  |  |  |  |  |  |  |  |  |
|  | GG | 327 (60.2) | 372 (53.1) | Ref. | 0.55616 | 176 (60.1) | 353 (53.1) | Ref. | 0.65149 | 594 (64.1) | 243 (64.3) | Ref. | 0.66059 |  |  |  |  |
|  | GC / CC | 216 (39.8) | 329 (46.9) | 1.08 (0.84-1.38) |  | 117 (39.9) | 312 (46.9) | 1.08 (0.78-1.5) |  | 332 (35.9) | 135 (35.7) | 0.94 (0.7-1.25) |  |  |  |  |  |
|  | Rec. |  |  |  |  |  |  |  |  |  |  |  |  |  |  |  |  |
|  | GG / GC | 524 (96.5) | 642 (91.6) | Ref. | **0.01419** | 283 (96.6) | 612 (92) | Ref. | 0.11492 | 888 (95.9) | 359 (95) | Ref. | 0.25662 |  |  |  |  |
|  | CC | 19 (3.5) | 59 (8.4) | 1.96 (1.12-3.42) |  | 10 (3.4) | 53 (8) | 1.8 (0.84-3.87) |  | 38 (4.1) | 19 (5) | 1.46 (0.77-2.76) |  |  |  |  |  |
|  | Overdom. |  |  |  |  |  |  |  |  |  |  |  |  |  |  |  |  |
|  | GG / CC | 346 (63.7) | 431 (61.5) | Ref. | 0.56671 | 186 (63.5) | 406 (61.1) | Ref. | 0.77659 | 632 (68.3) | 262 (69.3) | Ref. | 0.33649 |  |  |  |  |
|  | GC | 197 (36.3) | 270 (38.5) | 0.93 (0.72-1.19) |  | 107 (36.5) | 259 (38.9) | 0.95 (0.68-1.33) |  | 294 (31.7) | 116 (30.7) | 0.86 (0.64-1.16) |  |  |  |  |  |
|  | log Add. |  |  |  |  |  |  |  |  |  |  |  |  |  |  |  |  |
|  | X 0.1.2 | 543 (43.6) | 701 (56.4) | 1.16 (0.95-1.42) | 0.15453 | 293 (30.6) | 665 (69.4) | 1.14 (0.87-1.5) | 0.33177 | 926 (71) | 378 (29) | 1.01 (0.79-1.28) | 0.96535 |  |  |  |  |
| rs7962370 | Codom. |  |  |  |  |  |  |  |  |  |  |  |  |  |  |  |  |
|  | GG | 270 (71.8) | 382 (76.9) | Ref. | 0.17389 | 136 (74.7) | 359 (75.9) | Ref. | 0.53085 | 684 (73.7) | 281 (74.3) | Ref. | 0.99910 |  |  |  |  |
|  | GA | 98 (26.1) | 102 (20.5) | 0.73 (0.52-1.02) |  | 44 (24.2) | 101 (21.4) | 0.89 (0.56-1.43) |  | 224 (24.1) | 90 (23.8) | 0.99 (0.72-1.37) |  |  |  |  |  |
|  | AA | 8 (2.1) | 13 (2.6) | 0.85 (0.32-2.28) |  | 2 (1.1) | 13 (2.7) | 2.26 (0.41-12.54) |  | 20 (2.2) | 7 (1.9) | 1 (0.38-2.69) |  |  |  |  |  |
|  | Dom. |  |  |  |  |  |  |  |  |  |  |  |  |  |  |  |  |
|  | GG | 270 (71.8) | 382 (76.9) | Ref. | 0.06491 | 136 (74.7) | 359 (75.9) | Ref. | 0.82890 | 684 (73.7) | 281 (74.3) | Ref. | 0.97108 |  |  |  |  |
|  | GA / AA | 106 (28.2) | 115 (23.1) | 0.73 (0.53-1.02) |  | 46 (25.3) | 114 (24.1) | 0.95 (0.6-1.5) |  | 244 (26.3) | 97 (25.7) | 0.99 (0.73-1.36) |  |  |  |  |  |
|  | Rec. |  |  |  |  |  |  |  |  |  |  |  |  |  |  |  |  |
|  | GG / GA | 368 (97.9) | 484 (97.4) | Ref. | 0.86106 | 180 (98.9) | 460 (97.3) | Ref. | 0.30577 | 908 (97.8) | 371 (98.1) | Ref. | 0.98977 |  |  |  |  |
|  | AA | 8 (2.1) | 13 (2.6) | 0.92 (0.34-2.45) |  | 2 (1.1) | 13 (2.7) | 2.32 (0.42-12.83) |  | 20 (2.2) | 7 (1.9) | 1.01 (0.38-2.69) |  |  |  |  |  |
|  | Overdom. |  |  |  |  |  |  |  |  |  |  |  |  |  |  |  |  |
|  | GG / AA | 278 (73.9) | 395 (79.5) | Ref. | 0.06549 | 138 (75.8) | 372 (78.6) | Ref. | 0.59139 | 704 (75.9) | 288 (76.2) | Ref. | 0.96694 |  |  |  |  |
|  | GA | 98 (26.1) | 102 (20.5) | 0.73 (0.52-1.02) |  | 44 (24.2) | 101 (21.4) | 0.88 (0.55-1.4) |  | 224 (24.1) | 90 (23.8) | 0.99 (0.72-1.37) |  |  |  |  |  |
|  | log Add. |  |  |  |  |  |  |  |  |  |  |  |  |  |  |  |  |
|  | X 0.1.2 | 376 (43.1) | 497 (56.9) | 0.78 (0.59-1.04) | 0.09257 | 182 (27.8) | 473 (72.2) | 1.02 (0.68-1.53) | 0.93524 | 928 (71.1) | 378 (28.9) | 1 (0.75-1.32) | 0.97710 |  |  |  |  |

**Table F: Association analysis by logistic regression models for non-associated SNPs in *CACNA2D3* and *LRRK2* with leprosy *per se* in Rio de Janeiro (RIO).** Analysis performed using logistic regression models adjusted for: (1) sex and ancestry; (2) sex, age and ancestry. CO = controls; CA = cases; Ref. = reference; Codom. = codominant model; Dom. = dominant model; Rec. = recessive model; Overdom. = overdominant model; log Add = log. Additive model.

|  |  | **Rio de Janeiro^1^** | | | | **Rio de Janeiro^2^** | | | |
| --- | --- | --- | --- | --- | --- | --- | --- | --- | --- |
| **SNP** | **Model** | **CO (%)** | **CA (%)** | **OR (CI 95%)^1^** | **p-value^1^** | **CO (%)** | **CA (%)** | **OR (CI 95%)^2^** | **p-value^2^** |
| rs622326 | Codom. |  |  |  |  |  |  |  |  |
|  | CC | 314 (55.1) | 327 (48.4) | Ref. | 0.12453 | 171 (55.3) | 311 (48.4) | Ref. | 0.51265 |
|  | CT | 225 (39.5) | 273 (40.4) | 0.87 (0.67-1.12) |  | 119 (38.5) | 258 (40.1) | 0.92 (0.65-1.3) |  |
|  | TT | 31 (5.4) | 76 (11.2) | 1.41 (0.87-2.31) |  | 19 (6.1) | 74 (11.5) | 1.36 (0.7-2.66) |  |
|  | Dom. |  |  |  |  |  |  |  |  |
|  | CC | 314 (55.1) | 327 (48.4) | Ref. | 0.57969 | 171 (55.3) | 311 (48.4) | Ref. | 0.88577 |
|  | CT / TT | 256 (44.9) | 349 (51.6) | 0.93 (0.73-1.19) |  | 138 (44.7) | 332 (51.6) | 0.98 (0.7-1.36) |  |
|  | Rec. |  |  |  |  |  |  |  |  |
|  | CC / CT | 539 (94.6) | 600 (88.8) | Ref. | 0.08143 | 290 (93.9) | 569 (88.5) | Ref. | 0.28767 |
|  | TT | 31 (5.4) | 76 (11.2) | 1.51 (0.94-2.43) |  | 19 (6.1) | 74 (11.5) | 1.41 (0.74-2.7) |  |
|  | Overdom. |  |  |  |  |  |  |  |  |
|  | CC / TT | 345 (60.5) | 403 (59.6) | Ref. | 0.13679 | 190 (61.5) | 385 (59.9) | Ref. | 0.47895 |
|  | CT | 225 (39.5) | 273 (40.4) | 0.83 (0.65-1.06) |  | 119 (38.5) | 258 (40.1) | 0.89 (0.63-1.24) |  |
|  | log Add. |  |  |  |  |  |  |  |  |
|  | X 0.1.2 | 570 (45.7) | 676 (54.3) | 1.03 (0.85-1.25) | 0.76319 | 309 (32.5) | 643 (67.5) | 1.05 (0.8-1.36) | 0.74032 |
| rs3732748 | Codom. |  |  |  |  |  |  |  |  |
|  | GG | 152 (27.1) | 238 (35.6) | Ref. | 0.32240 | 77 (25.4) | 221 (34.9) | Ref. | 0.24505 |
|  | GA | 292 (52.1) | 323 (48.3) | 0.81 (0.62-1.07) |  | 166 (54.8) | 310 (48.9) | 0.72 (0.49-1.06) |  |
|  | AA | 116 (20.7) | 108 (16.1) | 0.84 (0.59-1.20) |  | 60 (19.8) | 103 (16.2) | 0.82 (0.51-1.32) |  |
|  | Dom. |  |  |  |  |  |  |  |  |
|  | GG | 152 (27.1) | 238 (35.6) | Ref. | 0.13572 | 77 (25.4) | 221 (34.9) | Ref. | 0.11499 |
|  | GA / AA | 408 (72.9) | 431 (64.4) | 0.82 (0.63-1.07) |  | 226 (74.6) | 413 (65.1) | 0.75 (0.52-1.08) |  |
|  | Rec. |  |  |  |  |  |  |  |  |
|  | GG / GA | 444 (79.3) | 561 (83.9) | Ref. | 0.78414 | 243 (80.2) | 531 (83.8) | Ref. | 0.94578 |
|  | AA | 116 (20.7) | 108 (16.1) | 0.96 (0.7-1.31) |  | 60 (19.8) | 103 (16.2) | 1.01 (0.67-1.52) |  |
|  | Overdom. |  |  |  |  |  |  |  |  |
|  | GG / AA | 268 (47.9) | 346 (51.7) | Ref. | 0.24982 | 137 (45.2) | 324 (51.1) | Ref. | 0.14333 |
|  | GA | 292 (52.1) | 323 (48.3) | 0.87 (0.68-1.1) |  | 166 (54.8) | 310 (48.9) | 0.79 (0.57-1.09) |  |
|  | log Add. |  |  |  |  |  |  |  |  |
|  | X 0.1.2 | 560 (45.6) | 669 (54.4) | 0.9 (0.76-1.08) | 0.24957 | 303 (32.3) | 634 (67.7) | 0.89 (0.7-1.12) | 0.32205 |
| rs7308720 | Codom. |  |  |  |  |  |  |  |  |
|  | CC | 407 (81.9) | 397 (77.1) | Ref. | 0.28979 | 216 (82.8) | 373 (77.1) | Ref. | 0.21845 |
|  | CG | 84 (16.9) | 106 (20.6) | 1.17 (0.84-1.64) |  | 42 (16.1) | 100 (20.7) | 1.42 (0.9-2.26) |  |
|  | GG | 6 (1.2) | 12 (2.3) | 1.98 (0.71-5.56) |  | 3 (1.1) | 11 (2.3) | 2.00 (0.47-8.52) |  |
|  | Dom. |  |  |  |  |  |  |  |  |
|  | CC | 407 (81.9) | 397 (77.1) | Ref. | 0.21837 | 216 (82.8) | 373 (77.1) | Ref. | 0.09241 |
|  | CG / GG | 90 (18.1) | 118 (22.9) | 1.23 (0.89-1.7) |  | 45 (17.2) | 111 (22.9) | 1.46 (0.94-2.29) |  |
|  | Rec. |  |  |  |  |  |  |  |  |
|  | CC / CG | 491 (98.8) | 503 (97.7) | Ref. | 0.20398 | 258 (98.9) | 473 (97.7) | Ref. | 0.37955 |
|  | GG | 6 (1.2) | 12 (2.3) | 1.92 (0.69-5.39) |  | 3 (1.1) | 11 (2.3) | 1.86 (0.44-7.89) |  |
|  | Overdom. |  |  |  |  |  |  |  |  |
|  | CC / GG | 413 (83.1) | 409 (79.4) | Ref. | 0.39927 | 219 (83.9) | 384 (79.3) | Ref. | 0.14972 |
|  | CG | 84 (16.9) | 106 (20.6) | 1.16 (0.83-1.62) |  | 42 (16.1) | 100 (20.7) | 1.40 (0.88-2.22) |  |
|  | log Add. |  |  |  |  |  |  |  |  |
|  | X 0.1.2 | 497 (49.1) | 515 (50.9) | 1.24 (0.93-1.65) | 0.14586 | 261 (35) | 484 (65) | 1.42 (0.95-2.12) | 0.08112 |
| rs10878434 | Codom. |  |  |  |  |  |  |  |  |
|  | GG | 286 (58.8) | 359 (60.6) | Ref. | 0.85149 | 146 (56.2) | 343 (61.4) | Ref. | 0.38507 |
|  | GA | 180 (37) | 208 (35.1) | 1.02 (0.78-1.34) |  | 103 (39.6) | 193 (34.5) | 0.90 (0.62-1.29) |  |
|  | AA | 20 (4.1) | 25 (4.2) | 0.84 (0.44-1.62) |  | 11 (4.2) | 23 (4.1) | 0.55 (0.23-1.31) |  |
|  | Dom. |  |  |  |  |  |  |  |  |
|  | GG | 286 (58.8) | 359 (60.6) | Ref. | 0.98108 | 146 (56.2) | 343 (61.4) | Ref. | 0.37712 |
|  | GA / AA | 200 (41.2) | 233 (39.4) | 1.00 (0.77-1.3) |  | 114 (43.8) | 216 (38.6) | 0.85 (0.6-1.21) |  |
|  | Rec. |  |  |  |  |  |  |  |  |
|  | GG / GA | 466 (95.9) | 567 (95.8) | Ref. | 0.58780 | 249 (95.8) | 536 (95.9) | Ref. | 0.21227 |
|  | AA | 20 (4.1) | 25 (4.2) | 0.84 (0.44-1.59) |  | 11 (4.2) | 23 (4.1) | 0.57 (0.24-1.35) |  |
|  | Overdom. |  |  |  |  |  |  |  |  |
|  | GG / AA | 306 (63) | 384 (64.9) | Ref. | 0.80404 | 157 (60.4) | 366 (65.5) | Ref. | 0.69061 |
|  | GA | 180 (37) | 208 (35.1) | 1.03 (0.79-1.35) |  | 103 (39.6) | 193 (34.5) | 0.93 (0.65-1.33) |  |
|  | log Add. |  |  |  |  |  |  |  |  |
|  | X 0.1.2 | 486 (45.1) | 592 (54.9) | 0.98 (0.78-1.23) | 0.86719 | 260 (31.7) | 559 (68.3) | 0.83 (0.62-1.13) | 0.23504 |
| rs3761863 | Codom. |  |  |  |  |  |  |  |  |
|  | CC | 182 (36) | 255 (37.4) | Ref. | 0.69871 | 95 (35.4) | 241 (37.4) | Ref. | 0.58035 |
|  | CT | 249 (49.3) | 311 (45.7) | 0.89 (0.68-1.17) |  | 143 (53.4) | 293 (45.5) | 0.95 (0.66-1.36) |  |
|  | TT | 74 (14.7) | 115 (16.9) | 0.94 (0.65-1.37) |  | 30 (11.2) | 110 (17.1) | 1.25 (0.73-2.13) |  |
|  | Dom. |  |  |  |  |  |  |  |  |
|  | CC | 182 (36) | 255 (37.4) | Ref. | 0.43335 | 95 (35.4) | 241 (37.4) | Ref. | 0.97384 |
|  | CT / TT | 323 (64) | 426 (62.6) | 0.9 (0.7-1.17) |  | 173 (64.6) | 403 (62.6) | 1.01 (0.71-1.42) |  |
|  | Rec. |  |  |  |  |  |  |  |  |
|  | CC / CT | 431 (85.3) | 566 (83.1) | Ref. | 0.96319 | 238 (88.8) | 534 (82.9) | Ref. | 0.31594 |
|  | TT | 74 (14.7) | 115 (16.9) | 1.01 (0.72-1.42) |  | 30 (11.2) | 110 (17.1) | 1.29 (0.78-2.11) |  |
|  | Overdom. |  |  |  |  |  |  |  |  |
|  | CC / TT | 256 (50.7) | 370 (54.3) | Ref. | 0.42845 | 125 (46.6) | 351 (54.5) | Ref. | 0.51189 |
|  | CT | 249 (49.3) | 311 (45.7) | 0.91 (0.71-1.16) |  | 143 (53.4) | 293 (45.5) | 0.89 (0.64-1.25) |  |
|  | log Add. |  |  |  |  |  |  |  |  |
|  | X 0.1.2 | 505 (42.6) | 681 (57.4) | 0.95 (0.8-1.14) | 0.60098 | 268 (29.4) | 644 (70.6) | 1.07 (0.84-1.37) | 0.59444 |
| rs7133914 | Codom. |  |  |  |  |  |  |  |  |
|  | GG | 458 (81.1) | 531 (76.8) | Ref. | 0.57176 | 249 (81.1) | 502 (76.6) | Ref. | 0.51564 |
|  | GA | 101 (17.9) | 149 (21.6) | 1.14 (0.84-1.55) |  | 55 (17.9) | 142 (21.7) | 1.26 (0.84-1.89) |  |
|  | AA | 6 (1.1) | 11 (1.6) | 1.42 (0.5-4.09) |  | 3 (1) | 11 (1.7) | 1.28 (0.29-5.70) |  |
|  | Dom. |  |  |  |  |  |  |  |  |
|  | GG | 458 (81.1) | 531 (76.8) | Ref. | 0.32821 | 249 (81.1) | 502 (76.6) | Ref. | 0.24984 |
|  | GA /AA | 107 (18.9) | 160 (23.2) | 1.16 (0.86-1.56) |  | 58 (18.9) | 153 (23.4) | 1.26 (0.85-1.88) |  |
|  | Rec. |  |  |  |  |  |  |  |  |
|  | GG / GA | 559 (98.9) | 680 (98.4) | Ref. | 0.53717 | 304 (99) | 644 (98.3) | Ref. | 0.78606 |
|  | AA | 6 (1.1) | 11 (1.6) | 1.39 (0.48-3.98) |  | 3 (1) | 11 (1.7) | 1.23 (0.28-5.44) |  |
|  | Overdom. |  |  |  |  |  |  |  |  |
|  | GG / AA | 464 (82.1) | 542 (78.4) | Ref. | 0.41038 | 252 (82.1) | 513 (78.3) | Ref. | 0.27038 |
|  | GA | 101 (17.9) | 149 (21.6) | 1.14 (0.84-1.54) |  | 55 (17.9) | 142 (21.7) | 1.26 (0.84-1.89) |  |
|  | log Add. |  |  |  |  |  |  |  |  |
|  | X 0.1.2 | 565 (45) | 691 (55) | 1.15 (0.88-1.51) | 0.29498 | 307 (31.9) | 655 (68.1) | 1.23 (0.85-1.78) | 0.26122 |

**Table G: Nominal p-values and false discovery rate (FDR)-adjusted p-values for SNP association analyses in populations of Rio de Janeiro (RIO) and Manaus (MAN).** The comparison model with the lowest AIC for each SNP was considered for the FDR analysis. 1 = association with leprosy *per se* (Tables S5 and S6); 2 = association with clinical form PB (multinomial analysis - Table 1); 3 = association with clinical form MB (multinomial analysis - Table 1).

| **SNP** | **Model** | **p-value RIO^1^** | **FDR p-value RIO^1^** | **p-value MAN^1^** | **FDR p-value MAN^1^** | **p-value RIO^2^** | **FDR p-value RIO^2^** | **p-value MAN^2^** | **FDR p-value MAN^2^** | **p-value RIO^3^** | **FDR p-value RIO^3^** | **p-value MAN^3^** | **FDR p-value MAN^3^** |
| --- | --- | --- | --- | --- | --- | --- | --- | --- | --- | --- | --- | --- | --- |
| rs1449325 | Overdominant | **0.00443** | **0.04235** | **0.000000119** | **0.000000595** | 0.0526 | 0.06575 | **0.0008** | **0.0040** | **0.0101** | **0.0341** | **0.0000845** | **0.0004225** |
| rs1375515 | Overdominant | **0.00770** | **0.04235** | 0.30106 | 0.50177 | **0.0452** | 0.06575 | 0.0660 | 0.1650 | **0.0327** | **0.0341** | 0.9606 | 0.96060 |
| rs13361189 | Recessive | **0.02133** | 0.05866 | 0.87473 | 0.97108 | **0.0341** | 0.06575 | 0.9329 | 0.9329 | **0.0187** | **0.0341** | 0.5622 | 0.94183 |
| rs11167514 | Recessive | **0.01419** | 0.05203 | 0.25662 | 0.50177 | **0.0114** | 0.05700 | 0.8244 | 0.9329 | **0.0241** | **0.0341** | 0.5651 | 0.94183 |
| rs7962370 | Dominant | 0.06491 | 0.14280 | 0.97108 | 0.97108 | 0.7949 | 0.79490 | 0.1686 | 0.2810 | **0.0341** | **0.0341** | 0.8359 | 0.96060 |
| rs622326 | Recessive | 0.08143 | 0.14929 | - | - | - | - | - | - | - | - | - | - |
| rs3732748 | Dominant | 0.13572 | 0.20056 | - | - | - | - | - | - | - | - | - | - |
| rs7308720 | log.Additive | 0.14586 | 0.20056 | - | - | - | - | - | - | - | - | - | - |
| rs10878434 | Recessive | 0.58780 | 0.58780 | - | - | - | - | - | - | - | - | - | - |
| rs3761863 | Dominant | 0.43335 | 0.47669 | - | - | - | - | - | - | - | - | - | - |
| rs7133914 | log.Additive | 0.29498 | 0.36053 | - | - | - | - | - | - | - | - | - | - |

**Table H: Association analysis for haplotype combinations of SNPs in *CACNA2D3* with leprosy *per se* in Rio de Janeiro (RIO).** Analysis performed by logistic regression models in the populations adjusted by sex and ancestry. CO = controls; CA = cases; Ref. = reference; Freq. = frequency.

| **Rio de Janeiro** | | | | | |
| --- | --- | --- | --- | --- | --- |
| **rs1449325** | **rs1375515** | **Freq. CO** | **Freq. CA** | **OR (CI 95%)** | **p-value** |
| C | T | 0.45418 | 0.43691 | Ref. | - |
| T | C | 0.39822 | 0.40842 | 0.89 (0.74-1.08) | 0.2458 |
| T | T | 0.11279 | 0.12125 | 1.01 (0.75-1.37) | 0.9490 |
| C | C | 0.03481 | 0.03343 | 0.97 (0.58-1.64) | 0.9150 |

**Table I:** **Association analysis for haplotype combinations of SNPs in *IRGM* with leprosy *per se* in Rio de Janeiro (RIO).** Analysis performed by logistic regression models in the populations adjusted by sex and ancestry. CO = controls; CA = cases; Ref. = reference; Freq. = frequency.

| **Rio de Janeiro** | | | | | |
| --- | --- | --- | --- | --- | --- |
| **rs13361189** | **rs11167514** | **Freq. CO.** | **Freq. CA.** | **OR (CI 95%)** | **p-value** |
| T | G | 0.77301 | 0.71194 | Ref. | - |
| C | C | 0.17553 | 0.24612 | 1.27 (1.02-1.58) | **0.0331** |
| T | C | 0.04734 | 0.03325 | 0.72 (0.46-1.12) | 0.1466 |

**FIGURES**

**Fig A: Description of the SNPs selection methodology.**

**
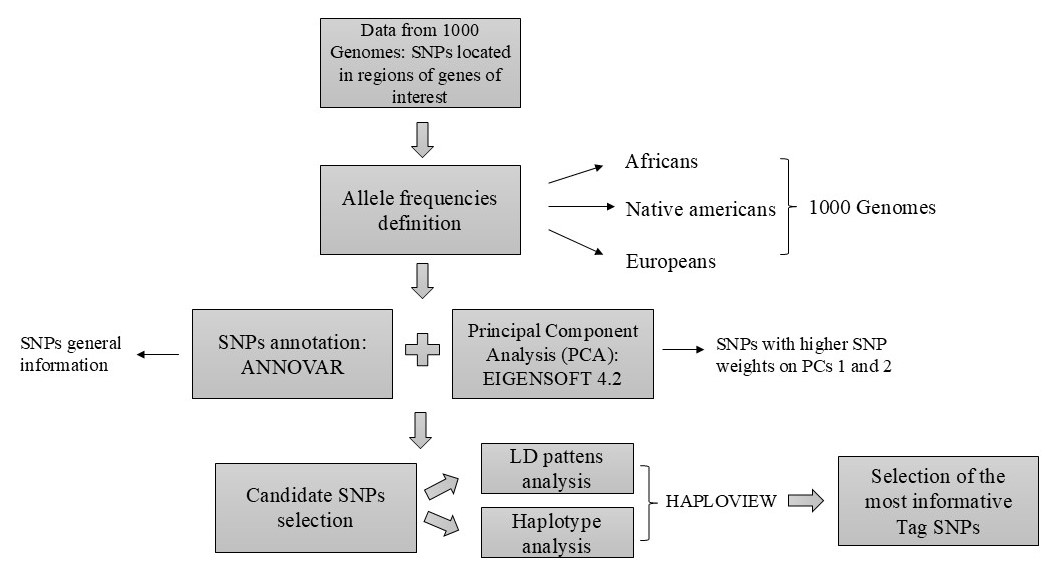
**

More information about SNPs selection methodology:

PCA consists of dimensional reduction of a set of variables observations, converting it into the smallest possible number of principal components (PCs) capable of recovering all the variability in analysis. Each PC is composed by a linear combination of all variables involved in analysis, but with different weights assigned to each one of them. These weights represent how much each of these variables is responsible for explaining the variability portion recovered in each PC. These PCs are numbered in descending order according to the percentages of variability recovered by each in the analysis. Thus, the variables with greater weights in PCs 1 and 2 are responsible for recovering higher percentages of variability in the analysis as a whole [1,2]. In this case, each PC is composed of a linear combination of genotypic values ​​for all SNPs located in and around the interest gene region. This type of analysis allows us to graphically access how these individuals are clustered according to each PC. Therefore, the SNPs responsible for recovering higher percentages of variability in analysis are those whose genotypic frequencies vary the most between clusters of individuals.

The variability for most loci located along the genome is often related to neutral evolutionary forces. However, when we analyze a particular gene, with a specific function, we assume that the existing variability is related to this. Therefore, we hypothesized that the SNPs with higher weights in PCs 1 and 2 have an important functional role related to the protein encoded by this gene. This is reasoned on the fact that only genetic variations capable of generating some phenotypic change can be influenced by selective environmental pressures. These are able to shape allelic and genotypic frequencies of loci related to the increase or decrease of an individual's adaptive advantage. This is directly related to ancestry profile of the individuals involved in analysis, as different populations are under different types of selective pressures. Therefore, we proposed that SNPs with a functional role in the proteins encoded by the genes of interest are more likely to exert a central biological effect on pathways involved in leprosy development.

References:

1. Ringnér M. What is principal component analysis?. Nature computational biology. 2008; 26(3):303-4.
2. Jolliffe IT, Cadima J. Principal component analysis: a review and recent developments. Phil. Trans. R. Soc. A. 2016; 374:20150202.

**Fig B: Principal Component Analysis (PCA) of the SNPs located from a region of 5,000 bp upstream and downstream of each candidate gene in the parental populations of the 1000 Genomes Project: *CACNA2D3* (A); *IRGM* (B) and; *LRRK2* (C).**

**A**
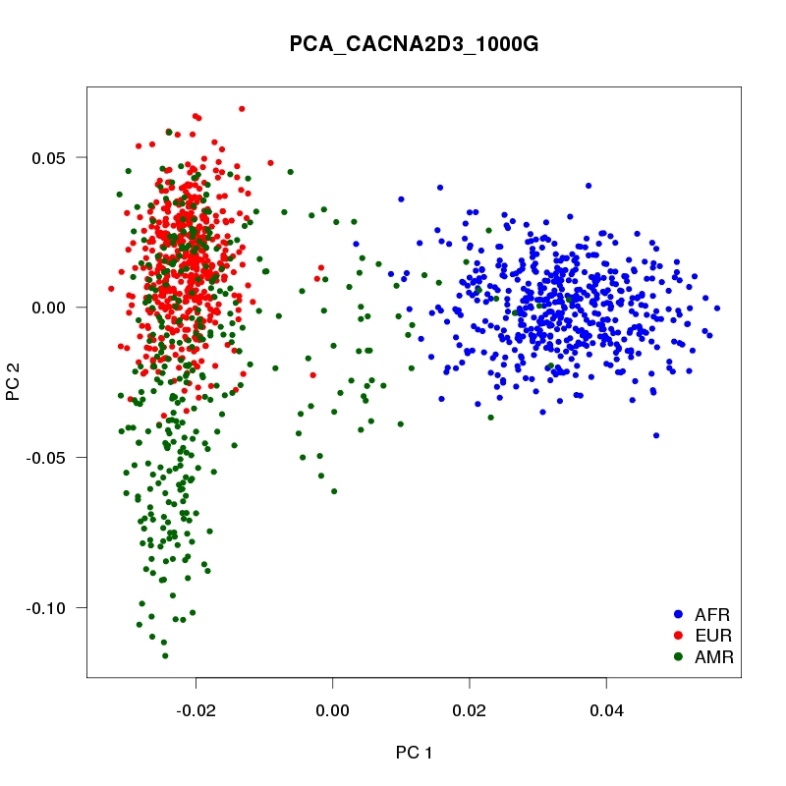


**B**
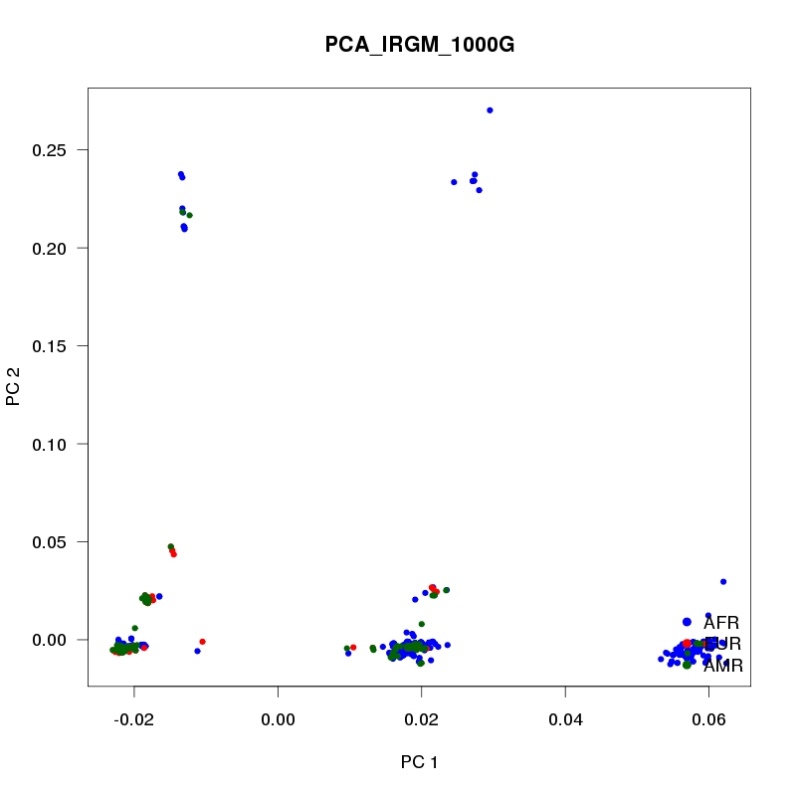


**C**
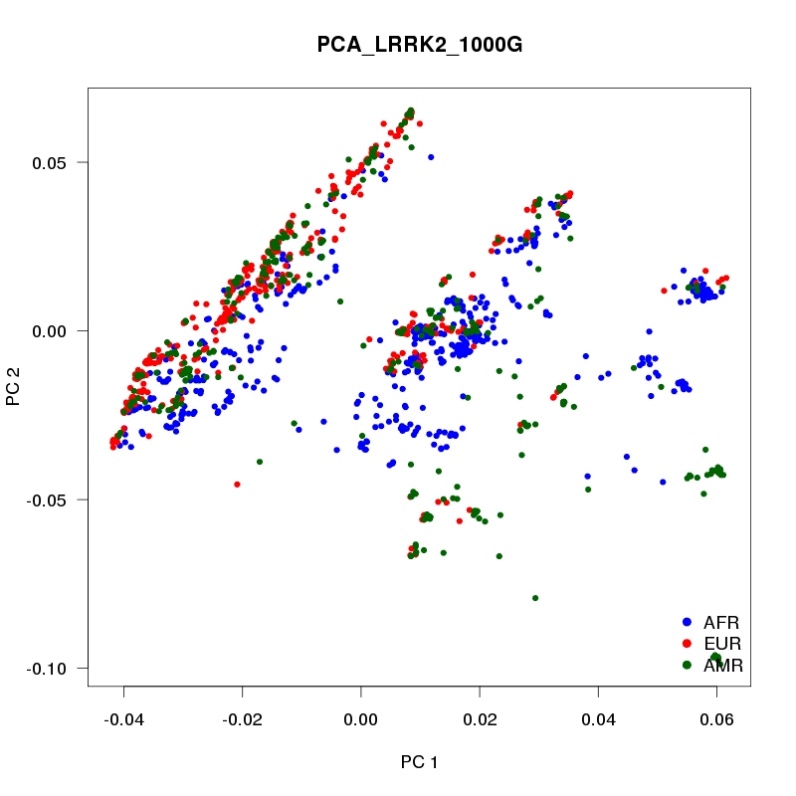


**Fig C: Linkage disequilibrium map between pre-selected SNPs in *CACNA2D3* region from annotation, PCA and frequency analysis for African (A) and European (B) parental populations of the 1000 Genomes Project.** Each square represents the LD pattern between two markers. Those with a value of r^2^ ≥ 0.8 (dark gray color) were considered in strong LD and those with a value of 0.6 < r^2^ < 0.8 (medium to dark gray color) in moderate LD. The numbers inside the squares are equal to the values of r^2^ multiplied by 100.

**A**


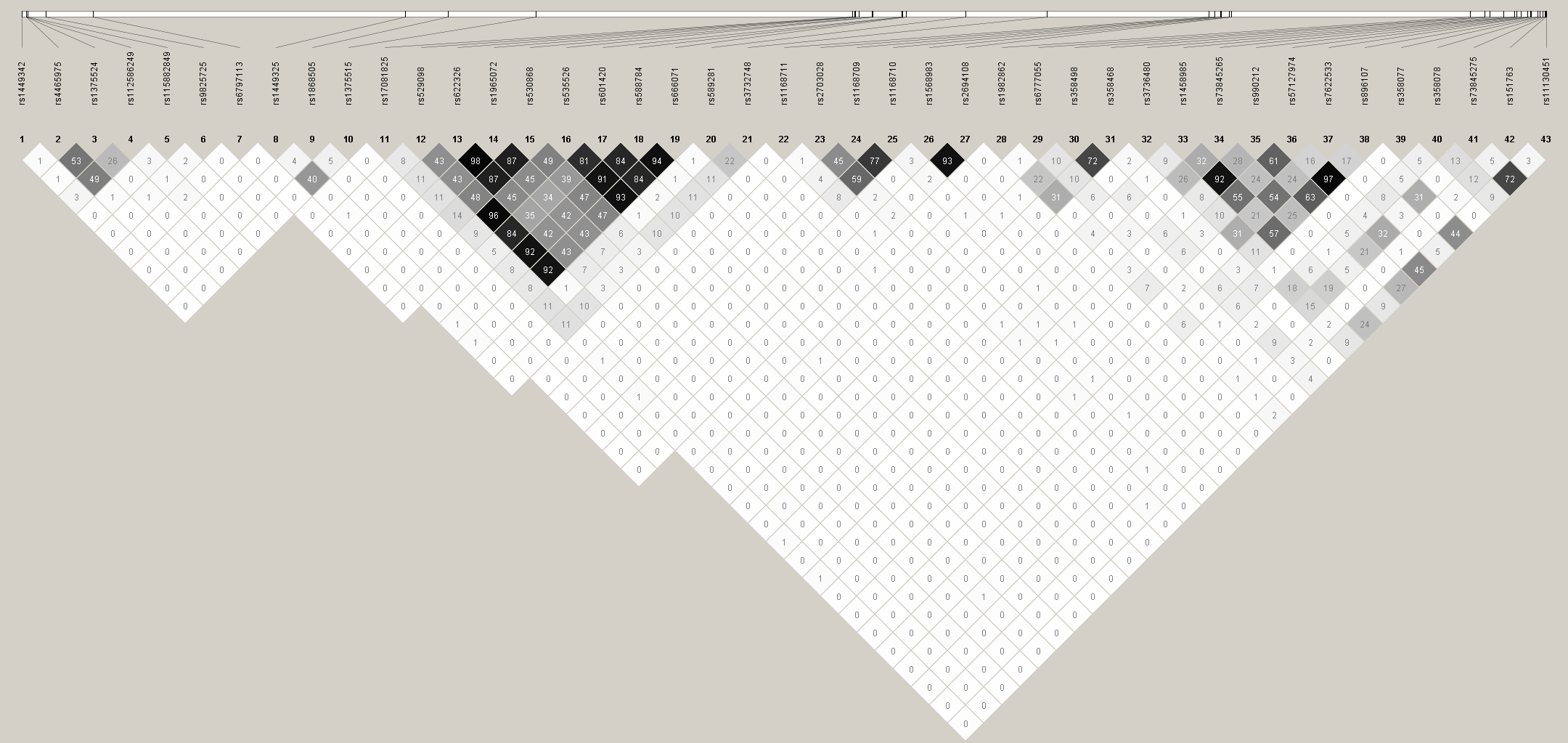


**B**


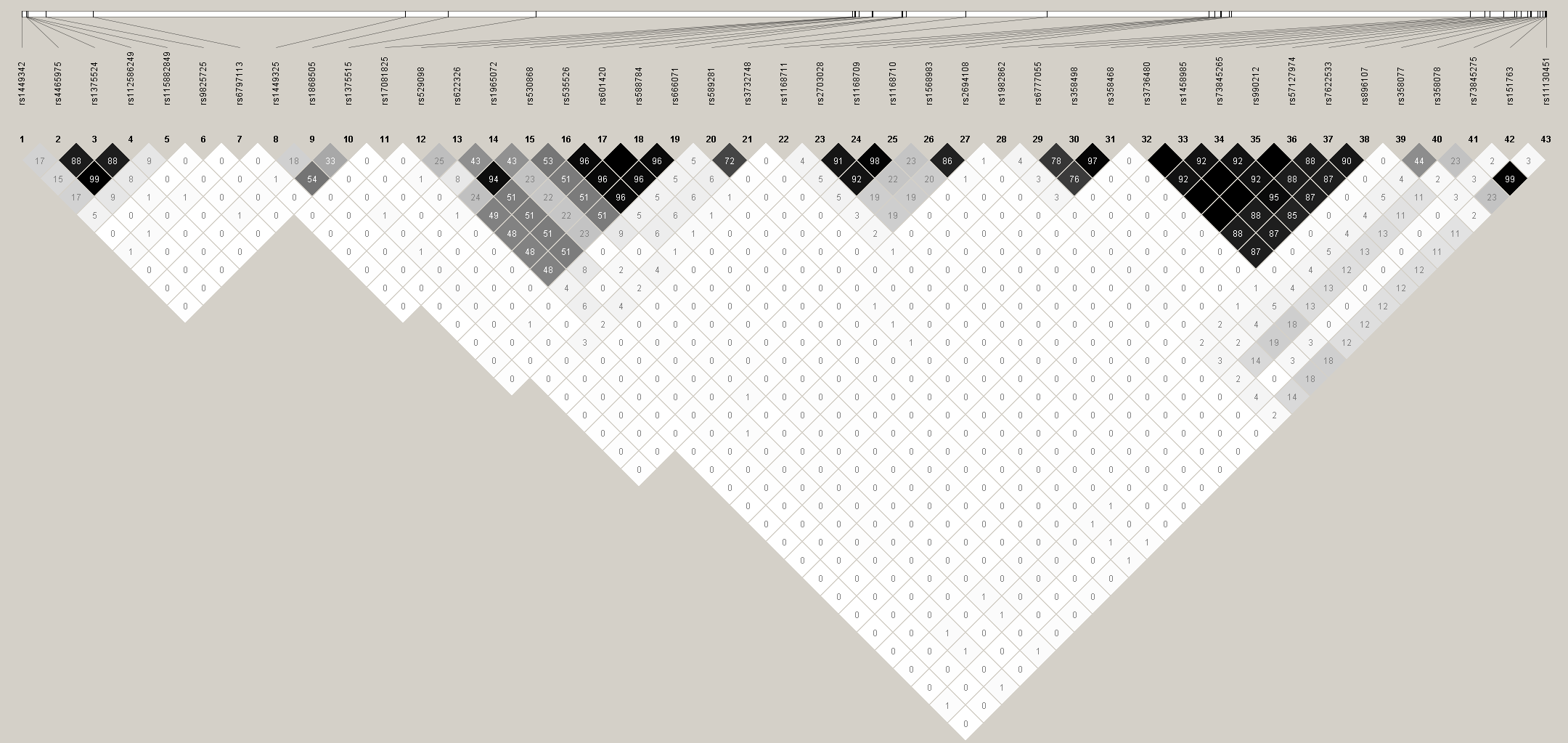


**Fig D: Linkage disequilibrium map between pre-selected SNPs in *IRGM* region from annotation, PCA and frequency analysis for African (A) and European (B) parental populations of the 1000 Genomes Project.** Each square represents the LD pattern between two markers. Those with a value of r^2^ ≥ 0.8 (dark gray color) were considered in strong LD and those with a value of 0.6 < r^2^ < 0.8 (medium to dark gray color) in moderate LD. The numbers inside the squares are equal to the values of r^2^ multiplied by 100.

**A**


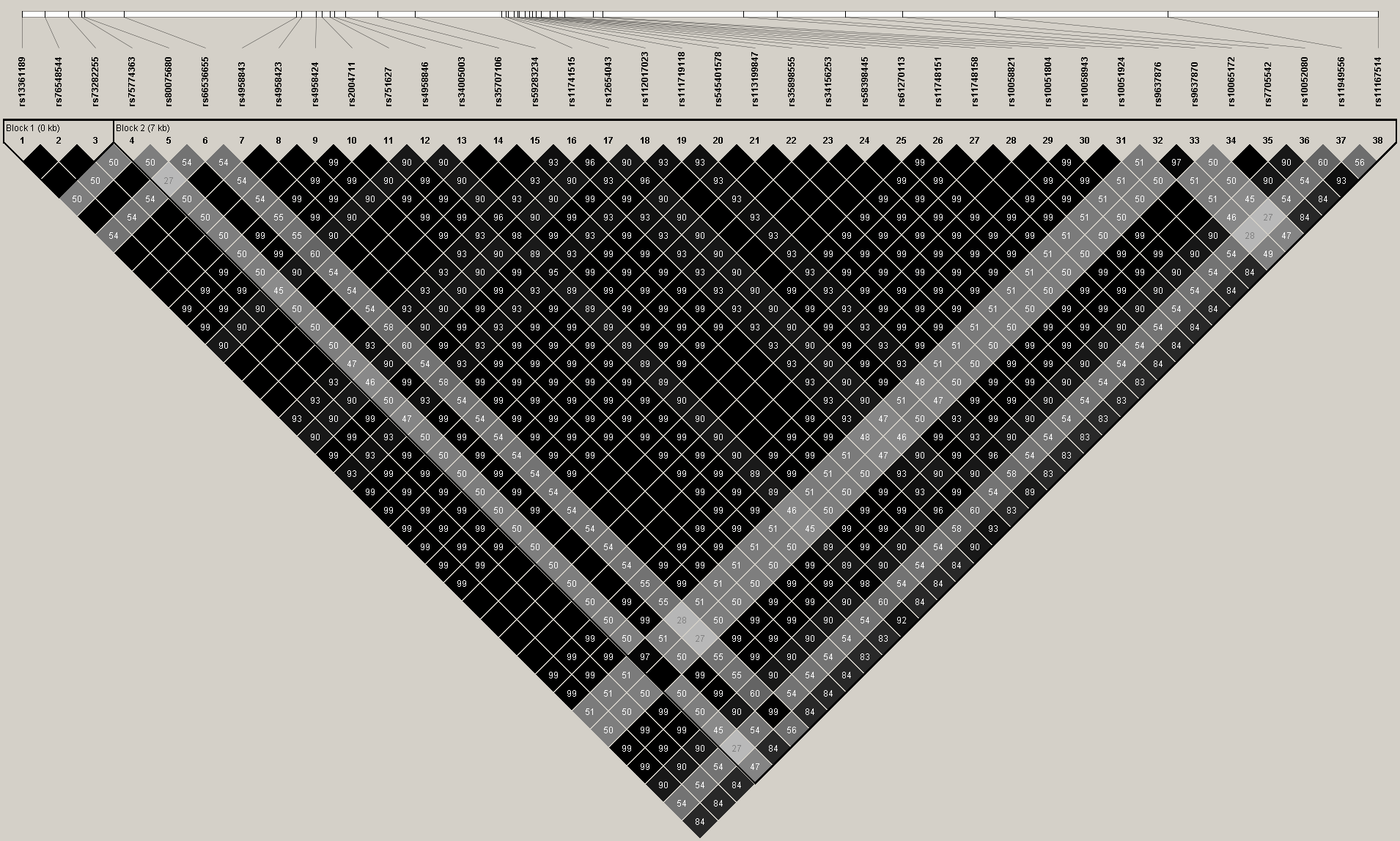


**B**


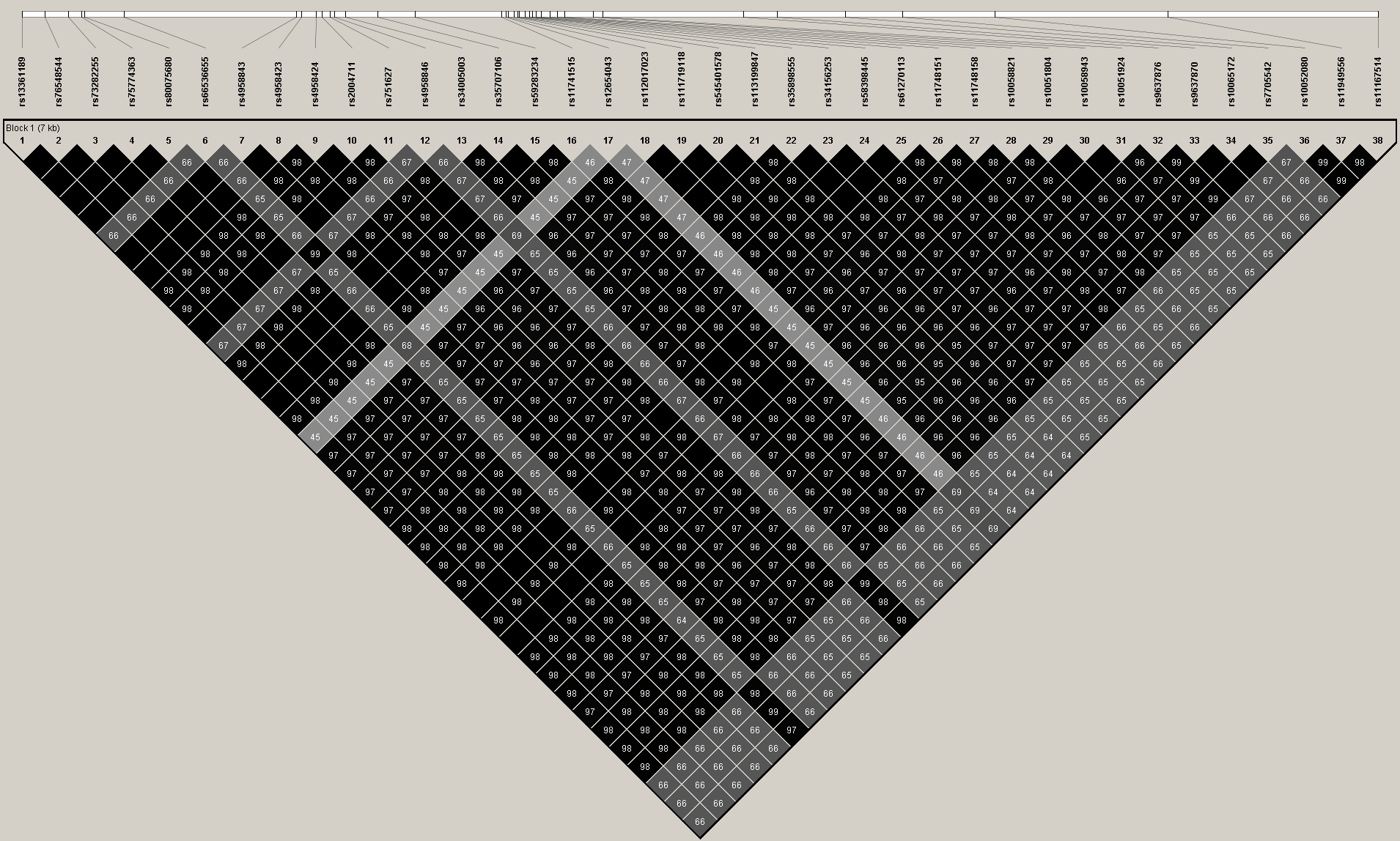


**Fig E: Linkage disequilibrium map between pre-selected SNPs in *LRRK2* region from annotation, PCA and frequency analysis for African (A) and European (B) parental populations of the 1000 Genomes Project.** Each square represents the LD pattern between two markers. Those with a value of r^2^ ≥ 0.8 (dark gray color) were considered in strong LD and those with a value of 0.6 < r^2^ < 0.8 (medium to dark gray color) in moderate LD. The numbers inside the squares are equal to the values of r^2^ multiplied by 100.

**A**


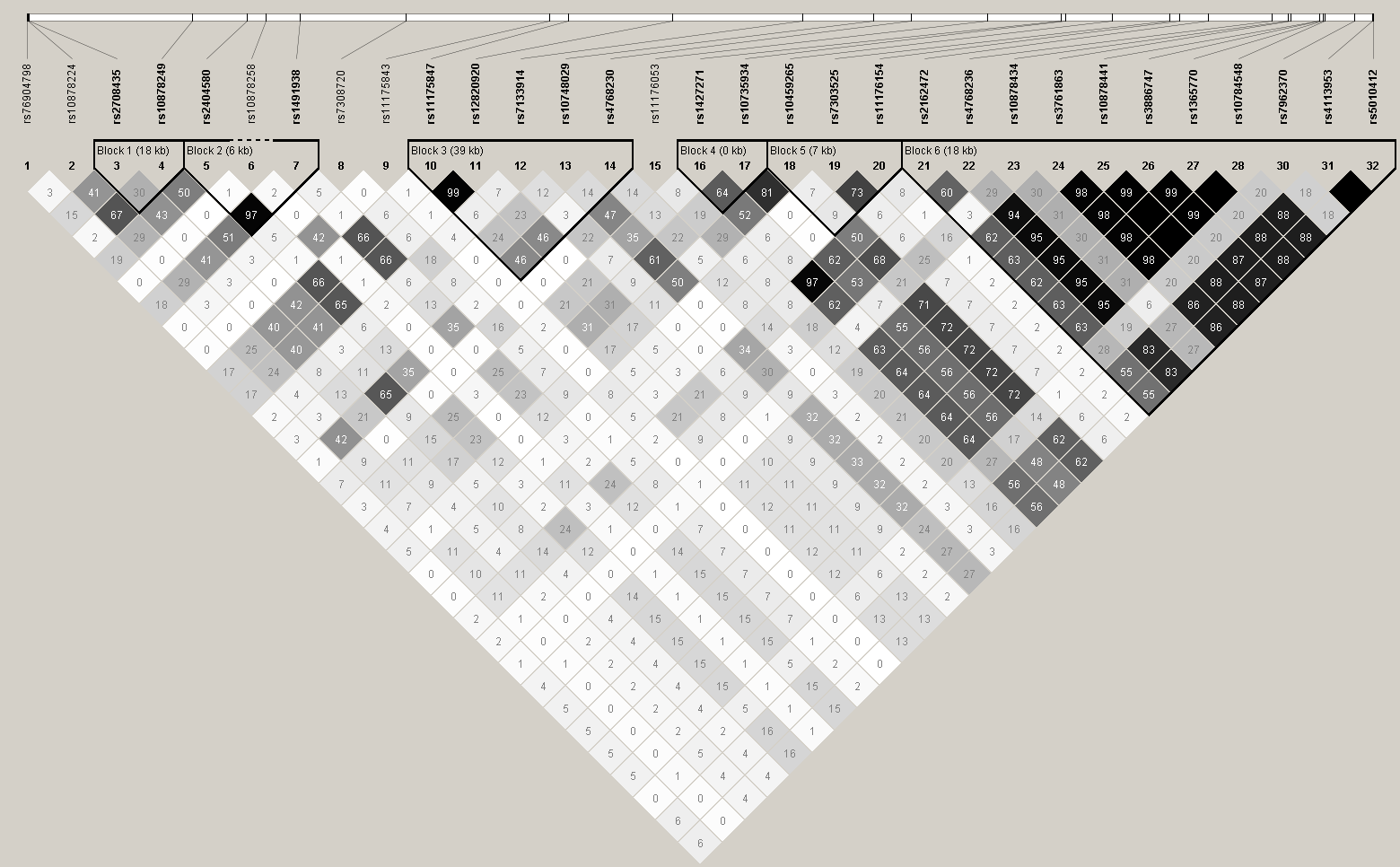


**B**


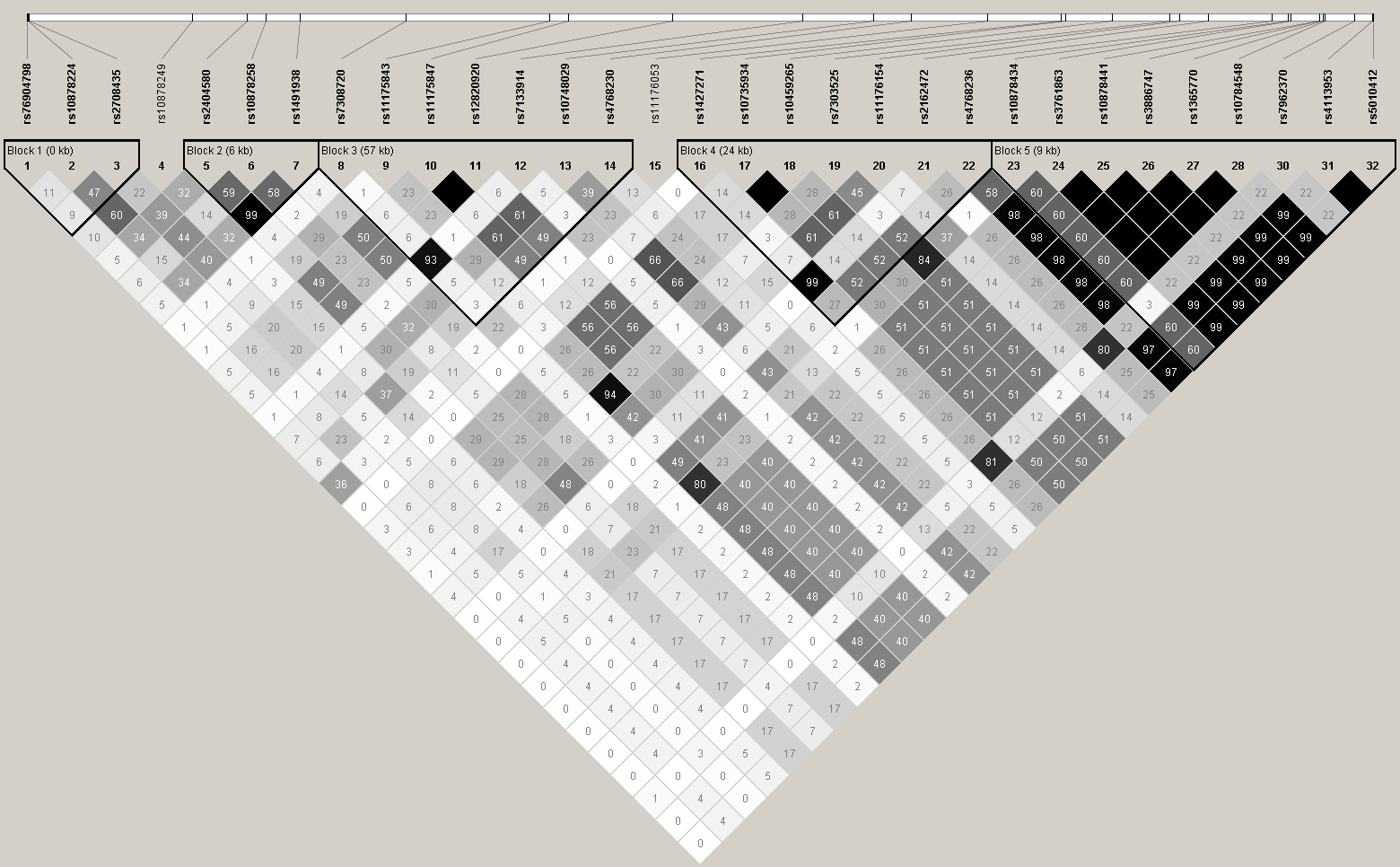


**Fig F: LD maps between selected SNPs in different genes for Rio de Janeiro (RIO) control cohort.** Each square represents the LD pattern for a pair of SNPs. Those with r2 ≥ 0.8 (dark gray color) were considered in strong LD and those with 0.6 ≤ r2 ≤ 0.79 (medium to dark gray color) in moderate LD. The numbers inside the squares are equal r2 values multiplied by 100. LD maps of SNPs located in *CACNA2D3* (A), *IRGM* (B) and *LRRK2* (C).

**A**
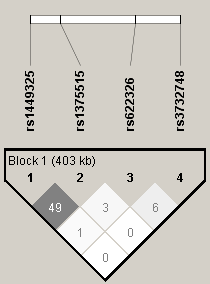
 **B**
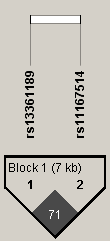
 **C**
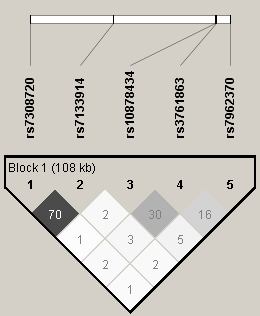


**Fig G: Expression analyses of *CACNA2D3* against** **rs1449325 genotypes in different models.** (A) Whole blood samples from leprosy *per se* patients against genotypes in the codominant model (B) same samples as in A, comparing PB (blue) and MB (orange) patients in the same model; (C) skin biopsies from leprosy *per se* patients against genotypes in the codominant model; (D) same samples as in C, comparing PB (blue) and MB (orange) patients in the same model; (E) whole blood samples from leprosy *per se* patients against genotypes in the overdominant model; (F) same samples as in E, comparing PB (blue) and MB (orange) patients in the same model; (G) skin biopsies from leprosy *per se* patients against genotypes in the overdominant model; (H) same samples as in G, comparing PB (blue) and MB (orange) patients in the same model; The means of the normalized expression values ​​of each group of genotypes were compared using: T-test with Welch correction (2 groups) and Brown-Forsythe and Welch Annova test followed by the Tamhane T2 multiple comparison test (3 groups), when the data followed a normal distribution; Mann-96-Whitney test (2 groups) and Kruskal-Wallis test followed by Dunn's post-test (3 groups), when the data were nonparametric. PB = paucibacillary; MB = multibacillary.

**
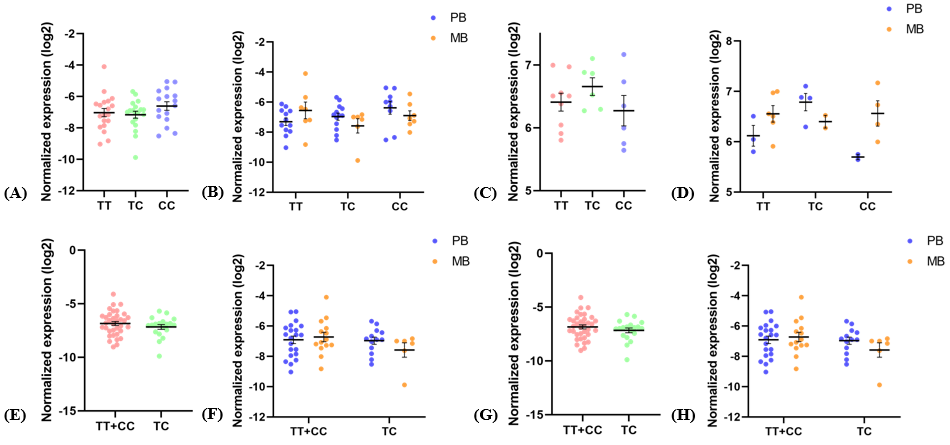
**

**Fig H: Expression analyses of *CACNA2D3* against** **rs1375515 genotypes.** (A) whole blood samples from leprosy *per se* patients against genotypes in the codominant model (B) same samples as in A, comparing PB (blue) and MB (orange) patients in the same model; (C) skin biopsies from leprosy *per se* patients against genotypes in the codominant model; (D) same samples as in C, comparing PB (blue) and MB (orange) patients in the same model; (E) whole blood samples from leprosy *per se* patients against genotypes in the overdominant model; (F) same samples as in E, comparing PB (blue) and MB (orange) patients in the same model; (G) skin biopsies from leprosy *per se* patients against genotypes in the overdominant model; (H) same samples as in G, comparing PB (blue) and MB (orange) patients in the same model; (I) whole blood samples from leprosy *per se* patients against genotypes in the dominant model; (J) same samples as in I, comparing PB (blue) and MB (orange) patients in the same model; (K) skin biopsies from leprosy *per se* patients against genotypes in the dominant model; (L) same samples as in K, comparing PB (blue) and MB (orange) patients in the same model. The means of the normalized expression values ​​of each group of genotypes were compared using: T-test with Welch correction (2 groups) and Brown-Forsythe and Welch Annova test followed by the Tamhane T2 multiple comparison test (3 groups), when the data followed a normal distribution; Mann-Whitney test (2 groups) and Kruskal-Wallis test followed by Dunn's post-test (3 groups), when data were nonparametric. * = significant difference between the expression levels of the compared groups. PB = paucibacillary; MB = multibacillary.

**
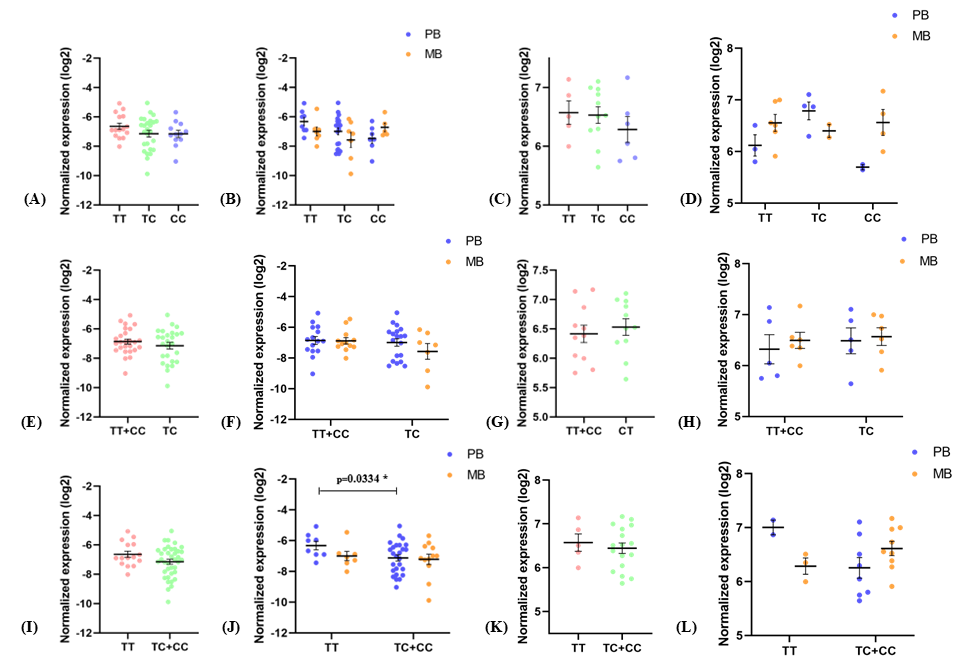
**

**Fig I: eQTL analysis from public database data of rs1375515 for *CACNA2D3* in monocytes stimulated with LPS for 6h.**

**Fig J: Expression analyses of *IRGM* against genotypes of associated SNPs.** (A) whole blood samples from leprosy *per se* patients against their rs13361189 genotypes in the dominant model; (B) same samples as in A, comparing PB (blue) and MB (orange) patients; (C) skin biopsies from leprosy *per se* patients against their rs11167514 genotypes in the dominant model; (D) same samples as in C, comparing PB (blue) and MB (orange) patients. The means of the normalized expression values ​​of each genotype group were compared using: Welch's t-test, when the data followed a normal distribution and Mann-Whitney test, when the data were nonparametric. PB = paucibacillary; MB = multibacillary.

**
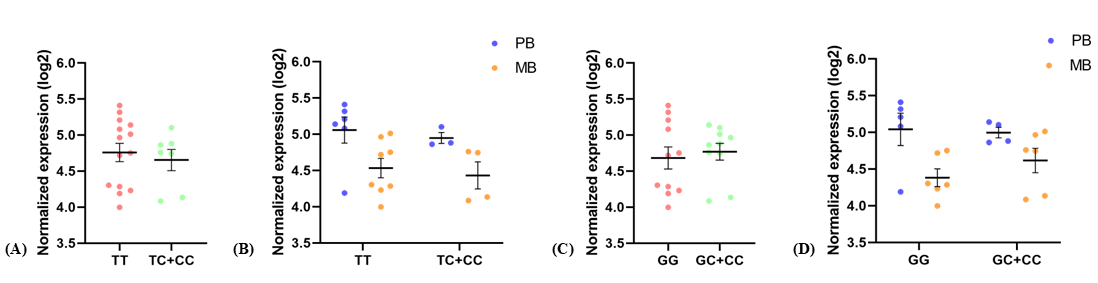
**

**Fig K: eQTL analyses from public databases of rs3761863 for *LRRK2* in: (A) unstimulated monocytes and; stimulated with (B) MDP for 6h.**

**(A)
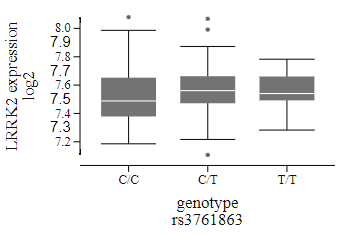
 (B)
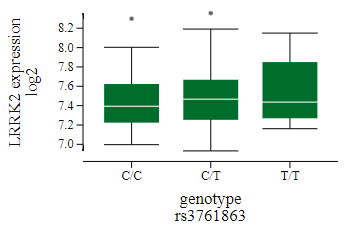
**

**Fig L:** **Possible impacts of variants in the *LRRK2* and *IRGM* in different scenarios of *M. leprae* infection in macrophages.** The symbols (+) and (-) represent activation/induction and inhibition/decrease, respectively. The arrows of different colors indicate relative changes when comparing the two scenarios, being: (green) positive regulation and; (red) negative regulation. The dashed arrows do not have a distinct meaning and are presented in this way only for organizational reasons. When recognized by macrophage membrane receptors, *M. leprae* is internalized and becomes a target for the autophagic machinery. Voltage-dependent calcium channels (Cav) act directly in the transport of Ca2+ into the cell. In a scenario favorable to *M. leprae* survival (A), LRRK2 interacts with NFAT, retaining it in the cytoplasm. Therefore, higher levels of this protein would be related to a lower translocation of these molecules to the nucleus, decreasing the expression of important cytokines, which would contribute to a less efficient cellular immune response. In addition, LRRK2 interacts with CD38 in the plasma membrane, and this complex is transported to the lysosomal membrane, where the kinase activity of LRRK2 is essential for the production of NAADP by CD38. This molecule activates the TPCN2 channel, responsible for Ca^2+^ transport from the interior of the lysosomes to the cytoplasm, leading to changes in calcineurin (CN). This acts by dephosphorylating TFEB, which contributes to a greater translocation of this molecule to the nucleus, positively regulating the expression of genes related to autophagy. Furthermore, Ca^2+^ also acts by phosphorylating CAMKKβ, which activates AMPK, inducing the initial stages of autophagy. However, LRRK2 activity also leads to morphological changes and decrease in acidity within lysosomes. Therefore, higher levels of LRRK2 and increase of its kinase activity are related to autophagy induction, but it is not completed properly, generating autophagosomes accumulation in the cytoplasm, which favors *M. leprae* survival*.* IRGM acts at different points in autophagy process, interacting with different proteins.In this scenario,lower expression of IRGM would be related to a negative autophagy regulation. In an unfavorable scenario for *M. leprae* survival (B), a decrease in the expression and kinase activity of LRRK2 would be related to a more efficient autophagic process, contributing to *M. leprae* clearance and to a more efficient cellular immune response. Furthermore, a greater expression of IRGM is related to a positive autophagy regulation, also contributing to *M. leprae* clearance. Created in BioRender. Espasandin, I. (2026) https://BioRender.com/sftpfq3 (A) and https://BioRender.com/q1hozlr (B).

**A**
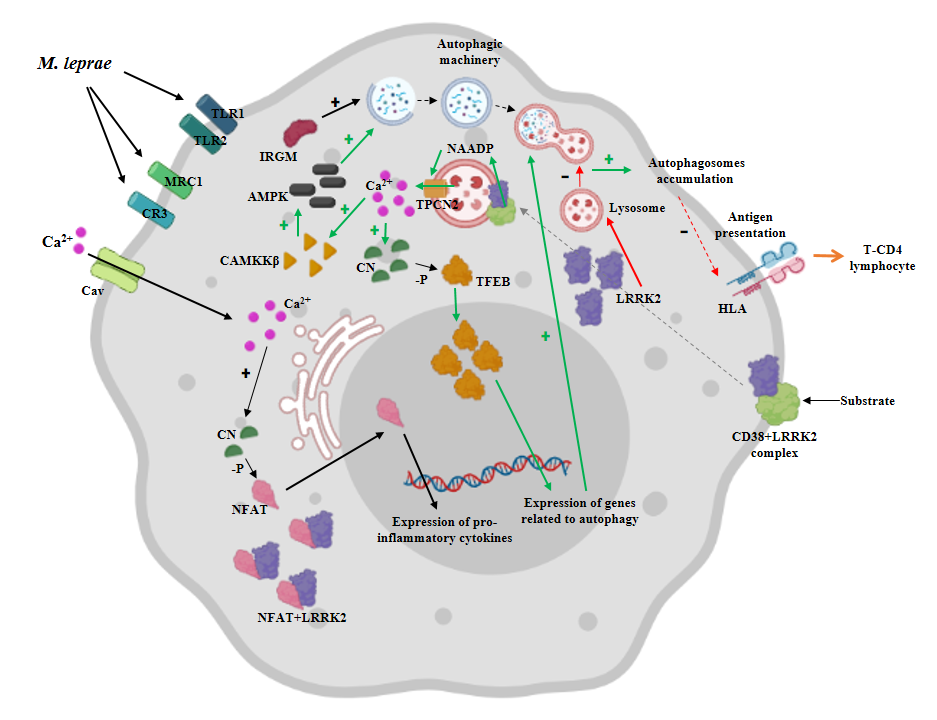


**B**
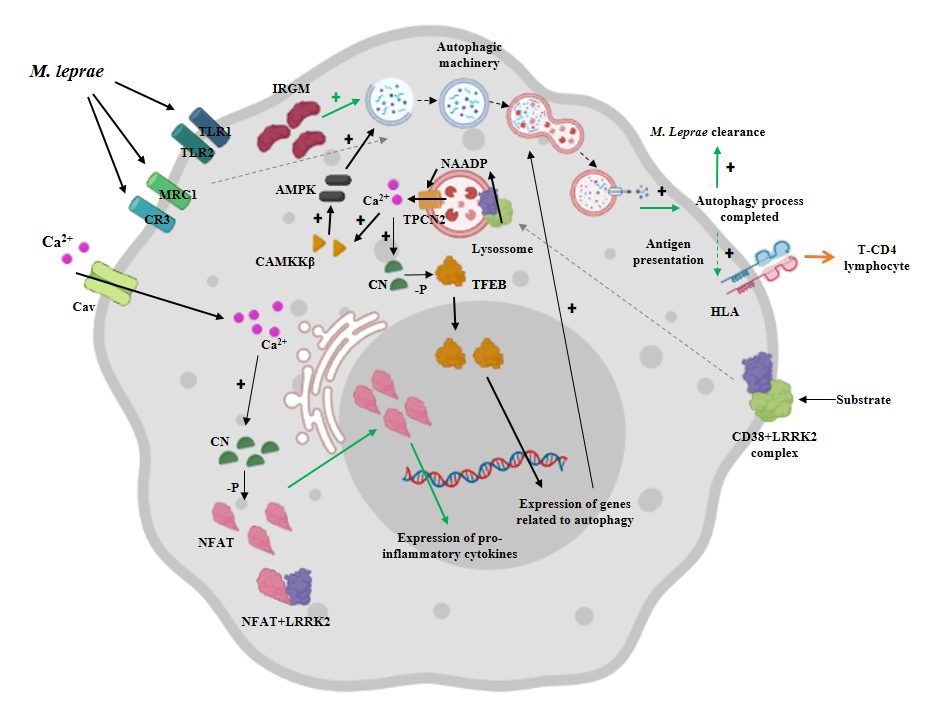

Supplement: S1 Text — Table A: Demographic characteristics of the populations studied. Table B: Information on the selected candidate SNPs located in the regions of CACNA2D3, IRGM and LRRK2. Table C: Demographic characteristics of the samples involved in the functional assays. Table D: Description of primers used in gene expression analysis. Table E: Association analysis by logistic regression models for associated SNPs in CACNA2D3, IRGM and LRRK2 with leprosy per se in Rio de Janeiro (RIO), Manaus (MAN) and Rondonópolis (ROO). Table F: Association analysis by logistic regression models for non-associated SNPs in CACNA2D3 and LRRK2 with leprosy per se in Rio de Janeiro (RIO). Table G: Nominal p-values and false discovery rate (FDR)-adjusted p-values for SNP association analyses in populations of Rio de Janeiro (RIO) and Manaus (MAN). Table H: Association analysis for haplotype combinations of SNPs in CACNA2D3 with leprosy per se in Rio de Janeiro (RIO). Table I: Association analysis for haplotype combinations of SNPs in IRGM with leprosy per se in Rio de Janeiro (RIO). Fig A: Description of the SNPs selection methodology. Fig B: Principal Component Analysis (PCA) of the SNPs located from a region of 5,000 bp upstream and downstream of each candidate gene in the parental populations of the 1000 Genomes Project: CACNA2D3 (A); IRGM (B) and; LRRK2 (C). Fig CLinkage disequilibrium map between pre-selected SNPs in CACNA2D3 region from annotation, PCA and frequency analysis for African (A) and European (B) parental populations of the 1000 Genomes Project. Fig D: Linkage disequilibrium map between pre-selected SNPs in IRGM region from annotation, PCA and frequency analysis for African (A) and European (B) parental populations of the 1000 Genomes Project. Fig E: Linkage disequilibrium map between pre-selected SNPs in LRRK2 region from annotation, PCA and frequency analysis for African (A) and European (B) parental populations of the 1000 Genomes Project. Fig F: LD maps between selected SNPs in di [file pntd.0014241.s001.docx]
