# Supplementary material for: Functional single nucleotide polymorphisms in CACNA2D3 and other autophagy-related genes are associated with leprosy among Brazilians
Source: PLoS Negl Trop Dis. 2026 Apr 27;20(4):e0014241. doi: 10.1371/journal.pntd.0014241 (PMC13120706; doi:10.1371/journal.pntd.0014241)
Supplement: S1 Checklist — Information on the STROBE Initiative is available at http://www.strobe-statement.org (DOCX) [file pntd.0014241.s002.docx]

STROBE Statement— Checklist of items that should be included in reports of case-control studies

1. **Title and abstract**

**(a) Indicate the study’s design with a commonly used term in the title or the abstract**

**(b) Provide in the abstract an informative and balanced summary of what was done and what was found**

**Manuscript page(s):** 1 (Title), 2 (Abstract)
**Relevant manuscript text:** Functional single nucleotide polymorphisms in *CACNA2D3* and other autophagy-related genes are associated with leprosy among Brazilians (Title).

“[...] A total of 3,480 individuals from three Brazilian populations were included in a case-control design.” (Abstract)

**Introduction**

1. **Background/rationale: Explain the scientific background and rationale for the investigation being reported.**

**Manuscript page(s):** 4–6
**Relevant manuscript text:** “[…] Therefore, the importance of autophagy in the pathophysiology of leprosy is already consolidated, thus highlighting the need to understand how polymorphisms in genes related to different regulatory pathways of this process can impact the outcome of this disease. Other genes related to autophagy and metabolism associated with leprosy and its clinical forms also were identified by GWAS such as LACC1, LRRK2, NOD2, among others.”

“[…] All together, these findings corroborate the importance of autophagy in the pathophysiology of leprosy, thus highlighting the need to understand how polymorphisms in genes related to different regulatory pathways of this process can impact the outcome of this disease. […]”

1. **Objectives: State specific objectives, including any prespecified hypotheses.**

**Manuscript page(s):** 6
**Relevant manuscript text:** “[…] Here we have further investigated the role of genes related to autophagy in leprosy outcome and clinical forms by investigating the association of candidate SNPs located in LRRK2, IRGM and CACNA2D3 genes. A potential functional role of the associated polymorphisms was also validated through gene expression analyses.”

**Methods**

1. **Study Design: Present key elements of study design early in the paper**

**Manuscript page(s):** 7-8.
**Relevant manuscript text:** “[…] The association study was conducted using a stepwise replication design, including three independent case-control populations with a total of 3,480 participants. First, a case-control analysis was conducted in a discovery sample from Rio de Janeiro (southeast Brazil), followed by a replication in Manaus (MAN, Amazonas State, North Brazil), and then replication in Rondonópolis (ROO, State of Mato Grosso, Midwest Brazil).”

1. **Setting: Describe the setting, locations, and relevant dates, including periods of recruitment, exposure, follow-up, and data collection**

**Manuscript page(s):** 7-8; 10-12.
**Relevant manuscript text:** “[…] A total of 1,338 were enrolled in Rio de Janeiro study. The cases group included 759 patients from Souza Araujo Out-Patient Unit (Fiocruz, Rio de Janeiro, Brazil) diagnosed with leprosy per se. Among these, 267 developed the paucibacillary (PB) form and 398 the multibacillary (MB) form. The control group was composed by 579 healthy individuals selected among bone marrow donors from the bank of the Cancer National Institute (INCA) (Rio de Janeiro-RJ, Brazil) [38]. The replication population of MAN consisted of 1,374 samples recruited at Fundação Hospitalar Alfredo da Matta (FUHAM, Manaus-AM, Brazil), with 407 cases and 967 controls. Patients were stratified as 130 PB and 277 MB, and healthy volunteers resident in the same endemic area as the cases were recruited for the control group in the same clinic (FUHAM), after a dermatological check-up [30]. The second replication population from ROO was composed of 768 individuals, including 411 cases and 357 controls. Patients from different public health institutions (primary care facilities), were divided as 96 PB and 310 MB. Healthy individuals were recruited in the same region during dermatological campaigns.”

“[...] blood samples from 55 patients recruited at the FIOCRUZ were collected before starting multidrug therapy and who did not develop reactional episodes, in RNA stabilizer tubes - PAXgene™ (PreAnalytix, QIAGEN, USA). Of these, 34 patients were diagnosed with PB leprosy and 21 with MB (Table S3).”

1. **Participants: Give the eligibility criteria, and the sources and methods of case ascertainment and control selection. Give the rationale for the choice of cases and controls.**

**Manuscript page(s):** 7-8
**Relevant manuscript text: “**The association study was conducted using a stepwise replication design, including three independent case-control populations with a total of 3,480 participants. [...] Controls were healthy individuals selected from blood donors or dermatological campaigns in the same endemic areas.”

1. **Variables: Clearly define all outcomes, exposures, predictors, potential confounders, and effect modifiers. Give diagnostic criteria, if applicable**

**Manuscript page(s):** 7-8
**Relevant manuscript text:** “[…] Leprosy clinical forms were determined using both the Ridley and Jopling classification method [35] and the World Health Organization guideline [36,37]. The demographic characteristics of the samples from the three populations used in the study are described in table S1.”

1. **Data sources/ measurement: For each variable of interest, give sources of data and details of methods of assessment (measurement). Describe comparability of assessment methods if there is more than one group**

**Manuscript page(s):** 8-10

**Relevant manuscript text:** “[…] The same analytical workflow was applied to CACNA2D3, IRGM, and LRRK2, following the methodology previously described by Bezerra et al. [38]. […]”

“[…] The DNA was extracted from whole blood samples by the salting out method [45] and all samples had their concentrations adjusted to 10–40 ng/μL. Samples were genotyped for all selected SNPs by real-time PCR with allelic discrimination using the TaqMan™ Genotyping Assays [46].”

“[…] The association of each SNP and haplotype with leprosy per se was determined using logistic regression models (codominant, dominant, recessive, superdominant and log-additive) adjusted for sex, age, and ancestry [38] (when these presented significant results from previous association analyses for such covariates with leprosy outcome).”

1. **Bias: Describe any efforts to address potential sources of bias**

**Manuscript page(s):** 13-14

**Relevant manuscript text:** “[…] Results of Rio cohort were similar when the covariate age was also included in the model (ORrecCC=1.51; p=0.03341; ORoverdTC= 0.65; p =0.01), despite the lower statistical power (Table S5).

1. **Study design: Explain how the study size was arrived at**

**Manuscript page(s):** 7-8

**Relevant manuscript text:** “[…] A total of 1,338 were enrolled in Rio de Janeiro study. The cases group included 759 patients from Souza Araujo Out-Patient Unit (Fiocruz, Rio de Janeiro, Brazil) diagnosed with leprosy per se. Among these, 267 developed the paucibacillary (PB) form and 398 the multibacillary (MB) form. The control group was composed by 579 healthy individuals selected among bone marrow donors from the bank of the Cancer National Institute (INCA) (Rio de Janeiro-RJ, Brazil) [38]. The replication population of MAN consisted of 1,374 samples recruited at Fundação Hospitalar Alfredo da Matta (FUHAM, Manaus-AM, Brazil), with 407 cases and 967 controls. Patients were stratified as 130 PB and 277 MB, and healthy volunteers resident in the same endemic area as the cases were recruited for the control group in the same clinic (FUHAM), after a dermatological check-up [30]. The second replication population from ROO was composed of 768 individuals, including 411 cases and 357 controls. Patients from different public health institutions (primary care facilities), were divided as 96 PB and 310 MB. Healthy individuals were recruited in the same region during dermatological campaigns.”

1. **Quantitative variables: Explain how quantitative variables were handled in the analyses. If applicable, describe which groupings were chosen and why**

**Manuscript page(s):** 9-10

**Relevant manuscript text:** “[…] The association of each SNP and haplotype with leprosy per se was determined using logistic regression models (codominant, dominant, recessive, superdominant and log-additive) adjusted for sex, age, and ancestry [38] (when these presented significant results from previous association analyses for such covariates with leprosy outcome).”

1. **Statistical methods:**

**(a) Describe all statistical methods, including those used to control for confounding**

**(b) Describe any methods used to examine subgroups and interactions**

**(c) Explain how missing data were addressed**

**(d) If applicable, explain how matching of cases and controls was addressed**

**(e) Describe any sensitivity analyses**

**Manuscript page(s):** 9-10

**Relevant manuscript text: “**The association of each SNP and haplotype with leprosy per se was determined using logistic regression models [...] adjusted for sex, age, and ancestry. [...] Multinomial regression models were used for analysis of leprosy clinical forms.”

**Results**

1. **Participants:**

**(a) Report numbers of individuals at each stage of study—eg numbers potentially eligible, examined for eligibility, confirmed eligible, included in the study, completing follow-up, and analysed**

**(b) Give reasons for non-participation at each stage**

**(c) Consider use of a flow diagram.**

**Manuscript page(s):** 7-8
**Relevant manuscript text:** “[…] A total of 1,338 were enrolled in Rio de Janeiro study. The cases group included 759 patients from Souza Araujo Out-Patient Unit (Fiocruz, Rio de Janeiro, Brazil) diagnosed with leprosy per se. Among these, 267 developed the paucibacillary (PB) form and 398 the multibacillary (MB) form. The control group was composed by 579 healthy individuals selected among bone marrow donors from the bank of the Cancer National Institute (INCA) (Rio de Janeiro-RJ, Brazil) [38]. The replication population of MAN consisted of 1,374 samples recruited at Fundação Hospitalar Alfredo da Matta (FUHAM, Manaus-AM, Brazil), with 407 cases and 967 controls. Patients were stratified as 130 PB and 277 MB, and healthy volunteers resident in the same endemic area as the cases were recruited for the control group in the same clinic (FUHAM), after a dermatological check-up [30]. The second replication population from ROO was composed of 768 individuals, including 411 cases and 357 controls. Patients from different public health institutions (primary care facilities), were divided as 96 PB and 310 MB. Healthy individuals were recruited in the same region during dermatological campaigns.”

1. **Descriptive data:**

**(a) Give characteristics of study participants (eg demographic, clinical, social) and information on exposures and potential confounders**

**(b) Indicate number of participants with missing data for each variable of interest**

**Manuscript page(s):** Table S1
**Relevant manuscript text:** Demographic characteristics including age, sex, clinical form and ancestry composition are described in Table S1.

1. **Outcome data: Report numbers in each exposure category, or summary measures of exposure.**

**Manuscript page(s):** Tables S5, Table 1
**Relevant manuscript text:** Summary measures for genotype frequencies, odds ratios and p-values are presented for each SNP in supplementary and main tables.

1. **Main results:**

**(a) Give unadjusted estimates and, if applicable, confounder-adjusted estimates and their precision (eg, 95% confidence interval). Make clear which confounders were adjusted for and why they were included**

**(b) Report category boundaries when continuous variables were categorized**

**(c) If relevant, consider translating estimates of relative risk into absolute risk for a meaningful time period**

**Manuscript page(s):** 13-15, Figure 1, Table 1; Tables S5
**Relevant manuscript text:** As shown in Figure 1 and Table S5, results of logistic regression models adjusted for sex and ancestry showed an association between SNP rs1449325 in CACNA2D3 and leprosy per se under recessive (ORrecCC=1.51; p=0.00476), and overdominant (ORoverdTC=0.70; p=0.00443) models in Rio de Janeiro population. These risk and protective associations were replicated, in the same recessive and overdominant models, in the populations of MAN (ORrecCC=3.06; p=1.44E-07; ORoverdTC= 0.37; p =1.19E-07) and ROO (ORrecCC=1.50; p=0.0240; ORoverdTC=0.67; p=0.0145). Results of Rio cohort were similar when the covariate age was also included in the model (ORrecCC=1.51; p=0.03341; ORoverdTC= 0.65; p =0.01), despite the lower statistical power (Table S5).

**17. Other analyses: Report other analyses done—e.g., analyses of subgroups and interactions, and sensitivity analyses.**

**Manuscript page(s):** 20-21

**Relevant manuscript text:** haplotype analyses, eQTL analyses, and gene expression correlations were performed and presented in the results and discussion.

**Discussion**

1. **Key results:** Summarise key results with reference to study objectives.

**Manuscript page(s):** 21-27
**Relevant manuscript text: “[…]**In this study, we observed that SNPs in *CACNA2D3*, *IRGM*, and *LRRK2* are associated with leprosy susceptibility and its clinical forms. Since these genes are involved, among other pathways, in autophagy regulation, it is reasonable to think that such polymorphisms are related to alterations in different points of this process […].”

**19. Limitations: Discuss limitations of the study, taking into account sources of potential bias or imprecision. Discuss both direction and magnitude of any potential bias.**

**Manuscript page(s):** 27
**Relevant manuscript text:** “[…] The understanding of results from association studies involving Brazilians becomes complex, because they make up a highly admixed population. We saw that associations found for several SNPs in Rio de Janeiro were not replicated in Manaus, which may be related to the considerable difference in genetic background of these populations, since leprosy is a complex disease. More studies are still needed to understand the exact biological role of some of these SNPs in leprosy, since not always an association can be explained by changes in transcriptional levels of a given gene, but also due to changes in the activity, stability or synthesis of the protein. encoded.”

**20. Interpretation: Give a cautious overall interpretation of results considering objectives, limitations, multiplicity of analyses, results from similar studies, and other relevant evidence.**

**Manuscript page(s):** 21-27
**Relevant manuscript text:** We observed consistent associations and proposed functional explanations, but highlighted the need for replication and functional validation.

**21. Generalisability: Discuss the generalisability (external validity) of the study results.**

**Manuscript page(s):** 27
**Relevant manuscript text:** “[…] The understanding of results from association studies involving Brazilians becomes complex, because they make up a highly admixed population. We saw that associations found for several SNPs in Rio de Janeiro were not replicated in Manaus, which may be related to the considerable difference in genetic background of these populations, since leprosy is a complex disease.”

**22. Funding: Give the source of funding and the role of the funders for the present study and, if applicable, for the original study on which the present article is based.**

**Manuscript page(s):** 27
**Relevant manuscript text:** Funding was provided by CAPES, CNPq, and FAPERJ. Funders had no role in study design, data collection, analysis, or publication decisions.
